# Does blood type affect the COVID-19 infection pattern?

**DOI:** 10.1371/journal.pone.0251535

**Published:** 2021-05-13

**Authors:** Mattia Miotto, Lorenzo Di Rienzo, Giorgio Gosti, Edoardo Milanetti, Giancarlo Ruocco

**Affiliations:** 1 Department of Physics, Sapienza University, Rome, Italy; 2 Center for Life Nano & Neuroscience, Istituto Italiano di Tecnologia, Rome, Italy; Columbia University, UNITED STATES

## Abstract

Among the many aspects that characterize the COVID-19 pandemic, two seem particularly challenging to understand: *i*) the great geographical differences in the degree of virus contagiousness and lethality that were found in the different phases of the epidemic progression, and, *ii*) the potential role of the infected people’s blood type in both the virus infectivity and the progression of the disease. A recent hypothesis could shed some light on both aspects. Specifically, it has been proposed that, in the subject-to-subject transfer, SARS-CoV-2 conserves on its capsid the erythrocytes’ antigens of the source subject. Thus these conserved antigens can potentially cause an immune reaction in a receiving subject that has previously acquired specific antibodies for the source subject antigens. This hypothesis implies a blood type-dependent infection rate. The strong geographical dependence of the blood type distribution could be, therefore, one of the factors at the origin of the observed heterogeneity in the epidemics spread. Here, we present an epidemiological deterministic model where the infection rules based on blood types are taken into account, and we compare our model outcomes with the exiting worldwide infection progression data. We found an overall good agreement, which strengthens the hypothesis that blood types do play a role in the COVID-19 infection.

## Introduction

The new infectious coronavirus disease 2019, called COVID-19, began to spread from China in December 2019 [1]. The most evident COVID-19 symptoms are pneumonia and respiratory failure, which reiterate the symptoms reported in the SARS (Severe Acute Respiratory Syndrome) epidemic of 2003 [2, 3]. The first cluster to clearly show these symptoms were patients from Wuhan, People’s Republic of China (WMHC) [2]. In early January 2020, scientists at the National Institute of Viral Disease Control and Prevention (IVDC) isolated the new virus for the first time from patients in Wuhan and found it to be a novel *β*-genus coronavirus, which has been named SARS-CoV-2 [4]. Currently, the outbreak has rapidly spread in many other countries. Hence, on 11 March 2020, the World Health Organization declared it a pandemic [5, 6].

Understanding the transmission dynamics of this infection plays a key role in assessing the diffusion potential that may be sustained in the future. In this context, models and simulations represent a powerful tool, which can be useful to study and monitor human and animal viral infections [7, 8]. These tools have become fundamental, especially during this pandemic, to evaluate the trade-off between cost and effectiveness of various social distancing strategies, and to enable policymakers to make the best decisions in the interest of public health [9]. Nonetheless, since each disease is characterized by specific biological rules, it is necessary to consider them to build an effective mathematical model able to describe real situations. As the virus spreads across the world, the pandemic has presented a similar pattern in all the countries that recorded a significant number of infections. The pattern is made up of a first phase, characterized by an exponential increase of infections, and a later phase, where the implementation of social distancing measures reduces the spread of the disease to a sub-exponential growth, generally followed by a gradual decrease of daily infections. Eventually, the number of daily infections becomes smaller than the daily recovered ones, thus allowing the number of the total infected individuals to decrease. Even if this general pattern has been reproduced around the world, the spread of the virus showed important local differences, mostly in the rate of the initial exponential spread. Given the complex nature of this historic event, it is extremely difficult to understand if these patterns are the consequence of geographical inhomogeneities or if these are spurious correlations that are caused by the singularity of the observed event. Indeed, some works underlined as in the early stage of the epidemics the recorded geographical pattern is characterized by the localization of most of the infection in temperate regions, distinguished by specific characteristics of temperature and humidity [10–13]. Other hypothesized co-morbidities that may explain local differences are hypertension, obesity, and age distribution, which are known to display heterogeneous local distributions [14, 15]. Also, the local history of past infections of different coronaviruses could contribute to the observed heterogeneity, due to cross-reactivity immunity effects [16, 17].

In particular, blood groups were recognized to influence susceptibility to certain viruses, including SARS-CoV-1 [18] and norovirus [19]. Blood group A and B glycosyltransferases also affect glycosylation in a large number of cell types, including epithelial cells in the respiratory tract and shed viral particles [20]. Recently, Zhao *et al.* [21] found that ABO blood groups presented a different risk to contract COVID-19 as a result of being exposed to SARS-CoV-2. Previously, for the similar coronavirus SARS-CoV responsible for SARS, Guillon *et al.* [22] showed experimentally that for SARS-CoV synthesized by cells that expressed the A histo-blood group antigen, the interaction between S protein and its membrane receptor, ACE2, could be blocked by anti-A blood group antibodies.

**Fig 1:**
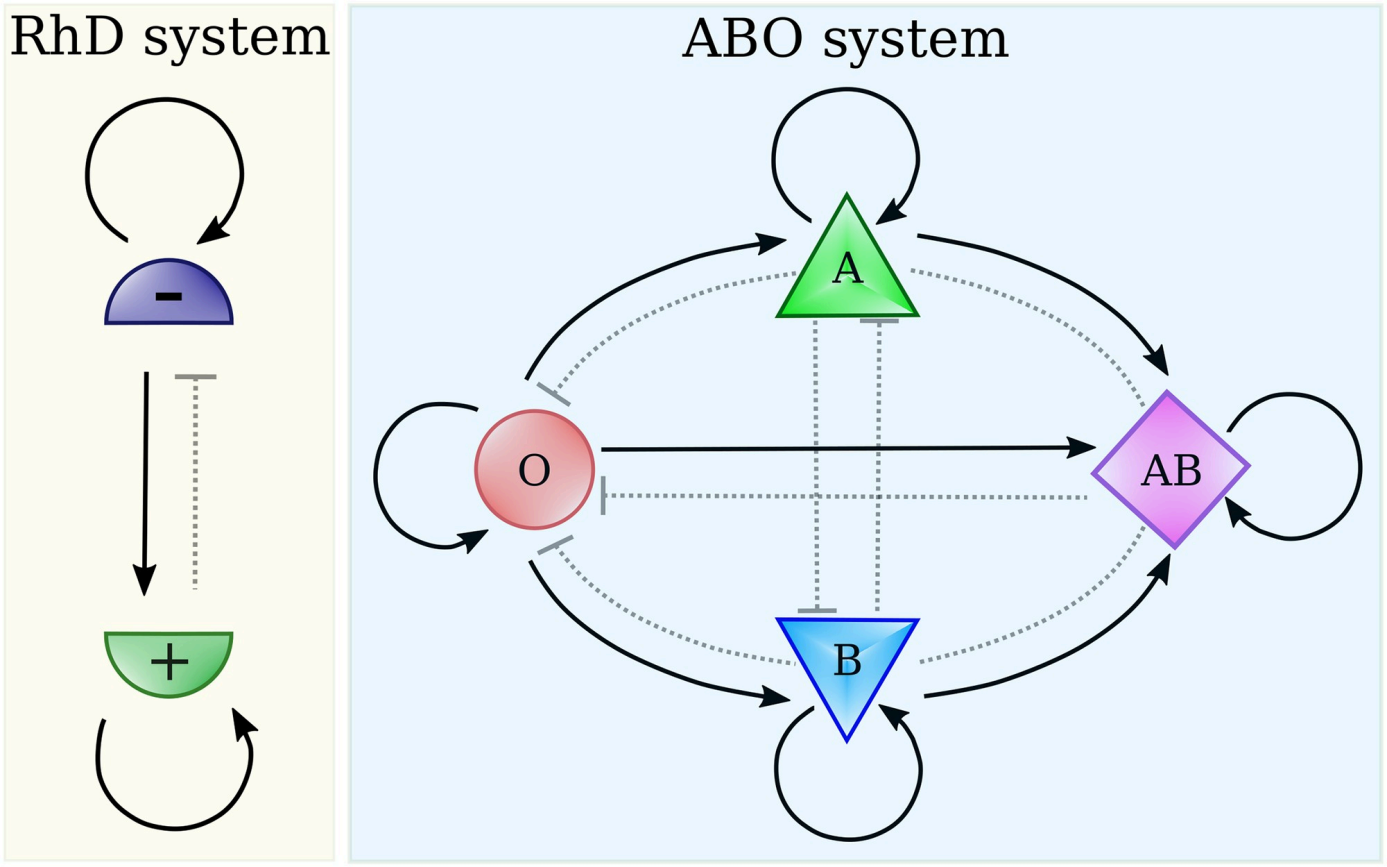
Scheme of the possible infection rules according to RhD blood types and ABO ones. Full line connection indicated the possibility of infection, *W*_*ij*_ = 1, while dotted connection its impossibility. In the RhD infection system, individuals of the same group can infect each other, people with the RhD- group can transmit the infection to people with RhD+ one, but not *viceversa*. The scenario of the ABO system is similar although richer. For instance, the O type can infect the *A* type, while the opposite is not possible.

Starting from these experimental results regarding the SARS-CoV spike, Breiman *et al.* [23] extended the hypothesis to the new SARS-CoV-2, suggesting that the different susceptibility of individuals with different ABO blood groups may have the same explanation. This new hypothesis, based on the infection rules schematically illustrated in Fig 1, may explain a part of the variability in the infection contagiousness among the countries of the world [23].

Here, we analyze the spread of the epidemic from a mathematical modeling perspective, taking into account the influence of blood type variability in different geographical areas. In particular, we provide a theoretical framework able to account for the differences in available data. The main purpose of this work is to verify whether the hypothesized blood group role in the transmission of the infection can be consistent with the acquired information on blood group distributions and infection data.

To this aim, we will first analyze available datasets of individuals infected by SARS-CoV-2, stratified by blood types. Then, we will deal with the contagion curves of a large set of countries and try to assess whether the present model could represent the real outcome of the COVID-19 pandemics. Specifically, we will first compare data collected in the Chinese regions of Wuhan and Shenzhen reported in [21], in Denmark [24], in the Ankara region (Turkey) [25], in New York City (NY, USA) [26], in Italy and Spain [27] and USA [28]. This allows for a direct comparison between our model predictions and real data. Then we move to analyze the early stages of the infection in different countries at a worldwide level. To take into account differences like lifestyle, climate, geographic location, and other factors that likely influence the epidemics rates as well, we identified four major areas, i.e. Europe, Asia, Africa, and South America.

## Materials and methods

### Infection data

To test the hypothesis that blood groups could impact the COVID-19 infection spread, we collected data from clinical observations of individuals found positive for SARS-CoV-2 for which also ABO blood group information was available. In Table 1, the ABO frequencies of positive patients (*d*_*i*_) and those of control populations (*f*_*i*_) are reported.

**Table 1:** Data used to determine the quantities plotted in Figs 2 and 3: ln(*d*_*i*_/*f*_*i*_) and $p_{T}^{\left(4\right)}$, with *d*_*i*_ being the fractions of infected having blood group *i*, *f*_*i*_ is the fraction of population with blood group *i* and $p_{T}^{\left(4\right)}$ represent the susceptibility of the population to become infected.

	Dataset	freq.	O	A	B	AB	Ref.
1	Wuhan Jinyintan Hospital	*f*_*i*_	0.3384	0.3216	0.2491	0.091	[21]
		*d*_*i*_	0.258	0.3775	0.2642	0.1003	
2	Renmin Hospital of Wuhan	*f*_*i*_	0.3384	0.3216	0.2491	0.091	[21]
		*d*_*i*_	0.2478	0.3982	0.2212	0.1327	
3	Shenzhen Third People Hospital	*f*_*i*_	0.3877	0.2877	0.2514	0.0732	[21]
		*d*_*i*_	0.2842	0.2877	0.2912	0.1368	
4	Central Hospital of Wuhan	*f*_*i*_	0.3384	0.3216	0.2491	0.091	[29]
		*d*_*i*_	0.2566	0.3925	0.2528	0.0981	
5	Denmark	*f*_*i*_	0.417	0.4238	0.1145	0.0447	[24]
		*d*_*i*_	0.3841	0.4441	0.1209	0.0509	
6	Hacettepe Hospital of Ankara	*f*_*i*_	0.3725	0.3804	0.1472	0.0999	[25]
		*d*_*i*_	0.2473	0.5699	0.1075	0.0753	
7	New York Presbyterian Hospital	*f*_*i*_	0.4814	0.3274	0.1491	0.0421	[26]
		*d*_*i*_	0.4575	0.3416	0.1701	0.0308	
8	Italy	*f*_*i*_	0.4709	0.3594	0.1299	0.0398	[27]
		*d*_*i*_	0.3749	0.4647	0.1090	0.0515	
9	Spain	*f*_*i*_	0.4863	0.4189	0.0684	0.0263	[27]
		*d*_*i*_	0.3755	0.4865	0.0916	0.0465	
10	USA (White non hispanic)	*f*_*i*_	0.4525	0.3974	0.1091	0.041	[28]
		*d*_*i*_	0.3779	0.451	0.1141	0.057	
11	USA (Black non hispanic)	*f*_*i*_	0.502	0.258	0.197	0.043	[28]
		*d*_*i*_	0.4791	0.2713	0.2171	0.0326	
12	USA (Asian non hispanic)	*f*_*i*_	0.3976	0.2777	0.2537	0.0709	[28]
		*d*_*i*_	0.2982	0.2807	0.3246	0.0965	
13	USA (Hispanic)	*f*_*i*_	0.5650	0.3110	0.0990	0.0250	[28]
		*d*_*i*_	0.6127	0.2941	0.0686	0.0245	

In particular, we consider:

- Four sets of data collected in three Wuhan hospitals and one Shenzhen hospital [21, 29]. The number of patients in the three sub-cases are 1775, 113,265, and 285, respectively. Notably, the abundance of each ABO blood group on the local population is reported as controls, allowing us to study the impact of local ABO phenotypic heterogeneities. Differences between infected and control frequencies were found with a higher level of statistical significance in the A and O groups.
- Another set was collected in recent work by Barnkob *et al.* [24] retrieved ABO group information for 7422 Danish individuals found positive to SARS-CoV-2 between 27 February 2020 and 30 July 2020. The reference ABO frequencies, *f*_*i*_ were instead obtained from about 2 million Danish people. In this case, statistical confidence is found for O,A, and AB groups (p-value <0.05), while B group is associated to a p-value of 0.091 (see [24]).
- Further data stratified by ABO blood groups are reported in [25] for 186 patients with a confirmed diagnosis of COVID-19 in the region of Ankara. The fraction of infected, *d*_*i*_ for each ABO blood group are reported in Table 1, together with the frequencies of the groups in a control sample of 1881 hospitalized individuals, whose blood groups were collected in the same period of time of the 186 infected cases. The reduced size of the sample assures statistical confidence only for A and O groups (p-value <0.05), with AB being the less trust-wort with a p-value of 0.364 (see [25]).
- Another work by Zietz *et al.* [26], collected information of ABO groups for 682 infected individuals tested in New York-Presbyterian/Columbia University Irving Medical Center (NYP/CUIMC). Statistical confidence is found for A, B, and O groups (p-value <0.05), while AB data has a p-value of 0.272 (see [26]).
- Finally, additional data was collected by two works that considered critically ill patients in Italy and Spain [27] and the United States [28]. Comparing blood frequencies of infected and control populations, a sub-representation of blood group O and an over-representation of group A was registered in both Italian and Spanish datasets, in agreement with the observations in Wuhan/Shenzhen and Denmark. Data collected by Leaf *et al.* [28] are stratified by both ABO blood groups and ethnicity.

As data of infections stratified by blood groups come from clinical observations taken in different hospitals, the statistical significance of the observed frequencies (especially on the less abundant blood groups) is not always guaranteed. As the amount of available data was limited, we opted not to discard data with low statistical significance; instead, we associate to all data a Poissonian error to be used as a weight in the analyses.

Moreover, we noted that only datasets 1 to 5 (see Table 1) were collected (i) not considering only severely ill patients and (ii) using not hospitalized patients as the control population. The remaining datasets may thus be affected by biases as, for instance, considering hospitalized patients affects the control blood group frequencies since pathologies are known to interest people with some blood groups more than others.

In addition to data stratified by blood groups, we collected data of the contagion by country from World Health Organization (WHO) Coronavirus Disease (COVID-19) Dashboard on date 12th of June 2020 [30]. To ensure statistical reliability, we selected only countries that had registered at least 2000 positive cases from the start of the epidemic. Requiring also to know the frequencies of both ABO and *RhD*±, we ended up with 78 countries, whose information is reported in Table 2.

**Table 2:** Percentages of blood groups (*f*_*i*_), susceptibility, $p_{T}^{\left(k\right)}$, and inverse characteristic time, *m*, of the exponential phase of the infection for the analyzed countries as derived for the fit to the observed data.

Country	Cluster	Code	*O*_+_	*A*_+_	*B*_+_	*AB*_+_	*O*_−_	*A*_−_	*B*_−_	*AB*_−_	$p_{T}^{\left(2\right)}$	$p_{T}^{\left(4\right)}$	*m*	Δ*m*	*R*^2^
Argentina	SA	AR	45.40	34.26	8.59	2.64	8.40	0.44	0.21	0.06	0.917	0.679	0.196	0.011	0.98
Armenia	AS	AM	29.00	46.30	12.00	5.60	2.00	3.70	1.00	0.40	0.934	0.618	0.143	0.025	0.97
Australia	AU	AU	40.00	31.00	8.00	2.00	9.00	7.00	2.00	1.00	0.846	0.660	0.223	0.005	0.99
Austria	EU	AT	30.00	37.00	12.00	5.00	6.00	7.00	2.00	1.00	0.866	0.612	0.216	0.033	0.98
Bahrain	AS	BH	48.48	19.35	22.61	3.67	3.27	1.33	1.04	0.25	0.945	0.635	0.356	0.054	0.97
Bangladesh	AS	BD	29.45	26.01	33.66	8.29	0.95	0.67	0.70	0.27	0.975	0.553	0.245	0.008	1.00
Belgium	EU	BE	38.00	34.00	8.60	4.10	7.00	6.00	1.50	0.80	0.870	0.647	0.209	0.019	0.99
Bolivia	SA	BO	51.53	29.45	10.11	1.15	4.39	2.73	0.54	0.10	0.928	0.680	0.148	0.012	0.95
Bosnia and Herzegovina	EU	BA	31.00	36.00	12.00	6.00	5.00	7.00	2.00	1.00	0.872	0.609	0.152	0.010	0.99
Brazil	SA	BR	36.00	34.00	8.00	2.50	9.00	8.00	2.00	0.50	0.843	0.653	0.272	0.015	0.98
Bulgaria	EU	BG	28.00	37.00	13.00	7.00	5.00	7.00	2.00	1.00	0.872	0.600	0.063	0.038	0.97
Cameroon	AF	CM	42.80	38.80	12.00	3.30	1.40	1.20	0.40	0.10	0.970	0.636	0.400	0.059	0.95
Canada	NA	CA	39.00	36.00	7.60	2.50	7.00	6.00	1.40	0.50	0.873	0.661	0.250	0.016	0.96
Chile	SA	CL	85.50	8.70	3.35	1.00	1.20	0.10	0.05	0.10	0.986	0.877	0.411	0.034	0.97
China	AS	CN	47.70	27.80	18.90	5.00	0.28	0.19	0.10	0.03	0.994	0.620	0.419	0.069	1.00
Colombia	SA	CO	61.30	26.11	2.28	1.47	5.13	2.70	0.70	0.31	0.919	0.754	0.218	0.015	0.97
Croatia	EU	HR	29.00	36.00	15.00	5.00	5.00	6.00	3.00	1.00	0.872	0.588	0.159	0.017	0.99
Cuba	SA	CU	45.80	33.50	10.20	2.90	3.60	2.80	1.00	0.20	0.930	0.654	0.220	0.020	0.95
Czechia	EU	CZ	27.00	36.00	15.00	7.00	5.00	6.00	3.00	1.00	0.872	0.583	0.287	0.029	0.98
Congo	AF	CD	59.50	21.30	15.20	2.40	1.00	0.30	0.20	0.10	0.984	0.685	0.123	0.010	0.99
Denmark	EU	DK	35.00	37.00	8.00	4.00	6.00	7.00	2.00	1.00	0.866	0.643	0.320	0.049	0.99
Dominican Republic	SA	DO	46.20	26.40	16.90	3.10	3.70	2.10	1.40	0.20	0.931	0.630	0.295	0.047	0.94
Ecuador	SA	EC	75.00	14.00	7.10	0.50	2.38	0.70	0.30	0.02	0.967	0.802	0.471	0.028	0.99
Egypt	AF	EG	52.00	24.00	12.40	3.80	5.00	2.00	0.60	0.20	0.928	0.672	0.158	0.029	0.97
El Salvador	SA	SV	62.00	23.00	11.00	1.00	1.00	1.00	0.70	0.30	0.971	0.706	0.148	0.007	0.98
Ethiopia	AF	ET	39.00	28.00	21.00	5.00	3.00	2.00	1.00	1.00	0.935	0.593	0.087	0.002	1.00
Finland	EU	FI	28.00	35.00	16.00	7.00	5.00	6.00	2.00	1.00	0.880	0.584	0.127	0.007	0.98
France	EU	FR	36.00	37.00	9.00	3.00	6.00	7.00	1.00	1.00	0.872	0.647	0.251	0.007	0.99
Germany	EU	DE	35.00	37.00	9.00	4.00	6.00	6.00	2.00	1.00	0.872	0.637	0.230	0.022	0.99
Ghana	AF	GH	53.80	17.60	18.30	2.80	4.50	1.30	1.30	0.20	0.928	0.668	0.091	0.013	0.98
Greece	EU	GR	37.40	32.90	11.00	3.70	7.00	5.00	2.00	1.00	0.872	0.631	0.282	0.063	0.94
Guinea	AF	GN	46.88	21.64	22.86	4.52	2.00	0.90	1.00	0.20	0.961	0.621	0.094	0.005	0.99
Honduras	SA	HN	57.50	27.00	7.80	2.50	2.70	1.70	0.60	0.20	0.951	0.702	0.128	0.015	0.98
Hungary	EU	HU	27.00	33.00	16.00	8.00	5.00	7.00	3.00	1.00	0.866	0.577	0.132	0.006	1.00
India	AS	IN	27.85	20.80	38.14	8.93	1.43	0.57	1.79	0.49	0.959	0.565	0.191	0.005	1.00
Indonesia	AS	ID	36.82	25.87	28.85	7.96	0.18	0.13	0.15	0.04	0.995	0.572	0.292	0.044	0.97
Iran	AS	IR	33.50	27.00	22.20	7.00	4.00	3.00	2.50	0.80	0.908	0.575	0.054	0.004	1.00
Iraq	AS	IQ	32.10	25.00	25.60	7.40	3.60	2.70	2.70	0.90	0.911	0.567	0.092	0.003	1.00
Ireland	EU	IE	47.00	26.00	9.00	2.00	8.00	5.00	2.00	1.00	0.866	0.672	0.307	0.018	1.00
Israel	AS	IL	32.00	34.00	17.00	7.00	3.00	4.00	2.00	1.00	0.910	0.582	0.180	0.012	1.00
Italy	EU	IT	39.00	36.00	7.50	2.50	7.00	6.00	1.50	0.50	0.872	0.661	0.389	0.020	0.99
Ivory Coast	AF	CI	46.50	22.50	22.50	4.30	2.00	1.00	1.00	0.20	0.960	0.619	0.073	0.016	0.97
Japan	AS	JP	29.90	39.80	19.90	9.90	0.15	0.20	0.10	0.05	0.995	0.570	0.150	0.023	0.99
Kazakhstan	AS	KZ	30.70	29.80	24.20	8.30	2.30	2.20	1.80	0.70	0.935	0.560	0.085	0.014	0.98
Kenya	AF	KE	45.60	25.20	21.28	4.20	1.80	1.00	0.90	0.02	0.964	0.614	0.277	0.045	0.98
Luxembourg	EU	LU	35.00	37.00	9.00	4.00	6.00	6.00	2.00	1.00	0.872	0.637	0.207	0.022	0.97
Malaysia	AS	MY	34.32	30.35	27.37	7.46	0.17	0.15	0.14	0.04	0.995	0.563	0.220	0.018	0.96
Mexico	SA	MX	59.09	26.23	8.53	1.73	2.73	1.21	0.40	0.08	0.958	0.708	0.225	0.031	0.97
Moldova	EU	MD	28.50	31.80	17.60	7.00	5.00	6.00	3.00	1.10	0.872	0.574	0.168	0.008	0.99
Morocco	AF	MA	42.30	29.80	14.30	4.10	4.50	3.10	1.50	0.40	0.914	0.625	0.176	0.009	1.00
Nepal	AS	NP	35.20	28.30	27.10	8.60	0.30	0.20	0.20	0.10	0.992	0.567	0.135	0.025	0.96
Netherlands	EU	NL	39.50	35.00	6.70	2.50	7.50	7.00	1.30	0.50	0.864	0.669	0.439	0.036	0.99
Nigeria	AF	NG	51.30	22.40	20.70	2.60	1.60	0.70	0.60	0.10	0.971	0.640	0.155	0.020	0.92
Macedonia	EU	MK	30.00	34.00	15.00	6.00	5.00	6.00	3.00	1.00	0.872	0.588	0.224	0.034	0.97
Norway	EU	NO	33.20	41.60	6.80	3.40	5.80	7.40	1.20	0.60	0.873	0.661	0.304	0.047	0.96
Pakistan	AS	PK	26.63	21.60	34.40	9.52	2.17	1.66	3.57	0.45	0.928	0.557	0.075	0.005	0.99
Peru	SA	PE	70.00	18.40	7.80	1.60	1.40	0.50	0.28	0.02	0.978	0.761	0.385	0.021	0.99
Philippines	AS	PH	45.90	22.90	24.90	5.97	0.10	0.10	0.10	0.03	0.997	0.608	0.353	0.027	0.95
Poland	EU	PL	31.00	32.00	15.00	7.00	6.00	6.00	2.00	1.00	0.872	0.594	0.233	0.022	0.99
Portugal	EU	PT	36.20	39.80	6.60	2.90	6.10	6.80	1.10	0.50	0.876	0.666	0.389	0.035	0.99
Romania	EU	RO	28.00	36.50	13.60	6.80	5.00	6.50	2.40	1.20	0.872	0.594	0.184	0.009	0.99
Russian Federation	EU	RU	28.00	30.00	20.00	7.00	4.90	5.80	3.20	1.10	0.872	0.565	0.185	0.004	1.00
Saudi Arabia	AS	SA	47.80	23.90	17.00	4.00	4.00	2.00	1.00	0.30	0.932	0.638	0.190	0.037	0.96
Serbia	EU	RS	31.92	35.28	12.60	4.20	6.08	6.72	2.40	0.80	0.866	0.610	0.143	0.004	1.00
Singapore	AS	SG	44.70	23.90	24.50	5.60	0.60	0.30	0.30	0.10	0.987	0.604	0.178	0.066	0.98
South Africa	AF	ZA	39.00	32.00	12.00	3.00	6.00	5.00	2.00	1.00	0.880	0.629	0.208	0.024	0.98
South Korea	AS	KR	27.90	33.87	26.92	10.98	0.10	0.13	0.08	0.02	0.997	0.548	0.195	0.011	0.97
Spain	EU	ES	35.00	36.00	8.00	2.50	9.00	7.00	2.00	0.50	0.849	0.652	0.377	0.011	0.99
Sudan	AF	SD	48.00	27.70	15.20	2.80	3.50	1.80	0.80	0.20	0.941	0.642	0.110	0.003	1.00
Sweden	EU	SE	32.00	37.00	10.00	5.00	6.00	7.00	2.00	1.00	0.866	0.625	0.303	0.010	0.99
Switzerland	EU	CH	35.00	38.00	8.00	4.00	6.00	7.00	1.00	1.00	0.872	0.650	0.241	0.044	0.98
Thailand	AS	TH	40.80	16.90	36.80	4.97	0.20	0.10	0.20	0.03	0.995	0.605	0.270	0.006	0.99
Turkey	AS	TR	29.80	37.80	14.20	7.20	3.90	4.70	1.60	0.80	0.902	0.596	0.201	0.036	0.98
Ukraine	EU	UA	32.00	34.00	15.00	5.00	5.00	6.00	2.00	1.00	0.880	0.597	0.229	0.016	0.99
United Arab Emirates	AS	AE	44.10	21.90	20.90	4.30	4.30	2.10	2.00	0.40	0.920	0.618	0.135	0.007	0.98
United Kingdom	EU	BG	38.00	32.00	8.00	3.00	9.00	7.00	2.00	1.00	0.846	0.653	0.230	0.008	0.99
United States	NA	US	37.40	35.70	8.50	3.40	6.60	6.30	1.50	0.60	0.872	0.649	0.306	0.016	0.97
Venezuela	SA	VE	58.30	28.20	5.60	1.90	3.70	1.80	0.40	0.10	0.944	0.721	0.171	0.029	0.84

#### Generalize SIR model

To describe the dynamics of the COVID-19 epidemics, we developed a generalized SIR (Susceptible-Infected-Recovered) model where the transmission of the infection depends on the blood types of the individuals. Indicating with *x*_*i*_, *y*_*i*_, and *z*_*i*_ the percentages of susceptible, infected and recovered individuals in the population having blood type *i* (e.g. O, A, B or AB), we can describe the evolution of the epidemics as

$$\begin{array}{ccc} & \frac{dx_{i}}{dt} & =-\beta x_{i}\;\sum_{j=1}^{k}W_{ij}y_{j} \end{array}$$ (1)

$$\frac{dy_{i}}{dt}=\beta x_{i}\;\sum_{j=1}^{k}W_{ij}y_{j}-\gamma \;y_{i}$$ (2)

$$\frac{dz_{i}}{dt}=\gamma \;y_{i}$$ (3)

where *β* and *γ* are the infection and recovering rates while *W*_*ij*_ = 1 if transmission from *i* to *j* is favored, *W*_*ij*_ = 0 otherwise (see Fig 1). A more complete discussion of the model and some particular cases, such as the standard SIR (*k* = 1) or a two sub-population model, can be found in the S1 File.

The set of 3*k* differential Eq (1), together with the initial conditions:

$$\begin{array}{ccc} & x_{i}\left(0\right) & =f_{i}x_{o}\approx f_{i}\\ & y_{i}\left(0\right) & =f_{i}y_{o}\\ & z_{i}\left(0\right) & =0 \end{array}$$ (4)

and the sum rule

$$\begin{array}{c} \sum_{i=1}^{k}f_{i}=1 \end{array}$$ (5)

allows one to work out the short time expansion for the infected person fraction on each of the *k* sub-populations:

$$\begin{array}{c} y_{i}\left(\tau \right)=y_{o}f_{i}\;e^{\pi_{i}^{\left(k\right)}\tau }\;\;\;\;\;\;\;\;\;\;\;\;\;i=1,\ldots ,k \end{array}$$ (6)

with

$$\begin{array}{c} \pi_{i}^{\left(k\right)}=\sum_{j}W_{ij}f_{j}-\rho =p_{i}^{\left(k\right)}-\rho , \end{array}$$ (7)

From those expressions, it follows that

$$\begin{array}{c} \ln\left(d_{i}/f_{i}\right)=K+p_{i}^{\left(k\right)}\tau^{*}\;\;\;\;\;\;\;\;\;\;\;\;\;i=1..k \end{array}$$ (8)

with *K* = ln *y*_*o*_−ρτ* not depending on *i*. We thus expect a linear relation between ln(*d*_*i*_/*f*_*i*_) and $p_{i}^{\left(k\right)}$.

It follows that the growth rate associated to subpopulation ‘i’ in the initial exponential phase depends on the sum of the frequencies of blood groups that can infect group ‘i’ (via the term ∑_*j*_ *W*_*ij*_ *f*_*j*_) and this can provoke a large variability in the onset of the epidemics in different geographical areas since the blood frequencies are highly heterogeneous.

The total number of infected people can be derived summing the percentages of infected individuals of each group:

$$\begin{array}{c} y_{T}\left(\tau \right)=\sum_{i}y_{i}\left(\tau \right)=y_{o}\;e^{\pi_{T}^{\left(k\right)}\tau }\;\;\;\;\;\;\;\;\;\;\;\;\;i=1,\ldots ,k \end{array}$$ (9)

with

$$\begin{array}{c} \pi_{T}^{\left(k\right)}=\sum_{i}\sum_{j}f_{i}W_{ij}f_{j}-\rho =p_{T}^{\left(k\right)}-\rho \end{array}$$ (10)

which implicitly defines $p_{T}^{\left(k\right)}$. This simple expression for the inverse of the characteristic time of the infection at its initial stage is the sum of two terms:, $\pi_{T}^{\left(k\right)}=p_{T}^{\left(k\right)}-\rho$.

The first one,

$$\begin{array}{c} p_{T}^{\left(k\right)}=\sum_{i}\sum_{j}f_{i}W_{ij}f_{j} \end{array}$$ (11)

depends only on the abundance of the sub-population ($\bar{f}$) and on the infection rules (*W*_*ij*_), the second (*ρ* = *γ*/*β*) on the overall recovery and infection rates of populations.

To study the global effect of the population composition on the progression of the infection, we concentrate on the term $p_{T}^{\left(k\right)}$ which acts as a “susceptibility”.

It is worth to note that, for any *k*, the susceptibility is maximum when $\bar{f}=\left(0\ldots 0,1,0,\ldots 0\right)$, $p_{T}^{\left(k\right)}=1$, i.e. when one sub-population fraction dominates, while it minimum when the sub-populations are all of the same size: $\bar{f}=\left(1/k,1/k\ldots 1/k\right)$. In the latter case, for the infection rules reported before, $p_{T}^{\left(k\right)}={\left(3/4\right)}^{\left(k/2\right)}$. Thus the susceptibility decreases on increasing the number of sub-populations and decreases on equalizing their abundances. Infection rules and compositions of the population are expected to shape the infection dynamics along with the usual infection and recovering rates.

## Results

The proposed system of equations (Eq 1) can be analytically solved in the small-time limit, i.e. when the epidemic is in the exponential phase. Detailed calculations are reported in the Methods and S1 File.

### Infection data stratified by blood type

To test the hypothesis that blood groups could impact the COVID-19 infection spread, we collected data from clinical observations of individuals found positive to SARS-CoV-2 for which also ABO blood group information was available. In Table 1, the ABO frequencies of positive patients (*d*_*i*_) in a certain geographical area and those of control populations (*f*_*i*_) in that area are reported.

Interestingly, our model predicts that the two quantities are related as

$$\begin{array}{c} \ln\left(d_{i}/f_{i}\right)=K+p_{i}^{\left(k\right)}\tau^{*}\;\;\;\;\;\;\;\;\;\;\;\;\;i=1..k \end{array}$$ (12)

with τ* is the specific (reduced) time when the data have been collected, *K* a constant not depending on *i*, and $p_{i}^{\left(k\right)}=\sum_{j}W_{ij}f_{j}$ measures of the susceptibility of group *i* to the infection. We thus expect a linear relation between ln(*d*_*i*_/*f*_*i*_) and $p_{i}^{\left(k\right)}$. A detailed derivation of the presented relationship is provided in the Methods section and further discussed in the S1 File.

At first, we considered the Wuhan/Shenzhen data. In the papers by Zhao et al. [21] and Li et al. [29] data of the COVID-19 contagion are reported, stratified by ABO blood type (no info on the RhD± type are given).

**Fig 2:**
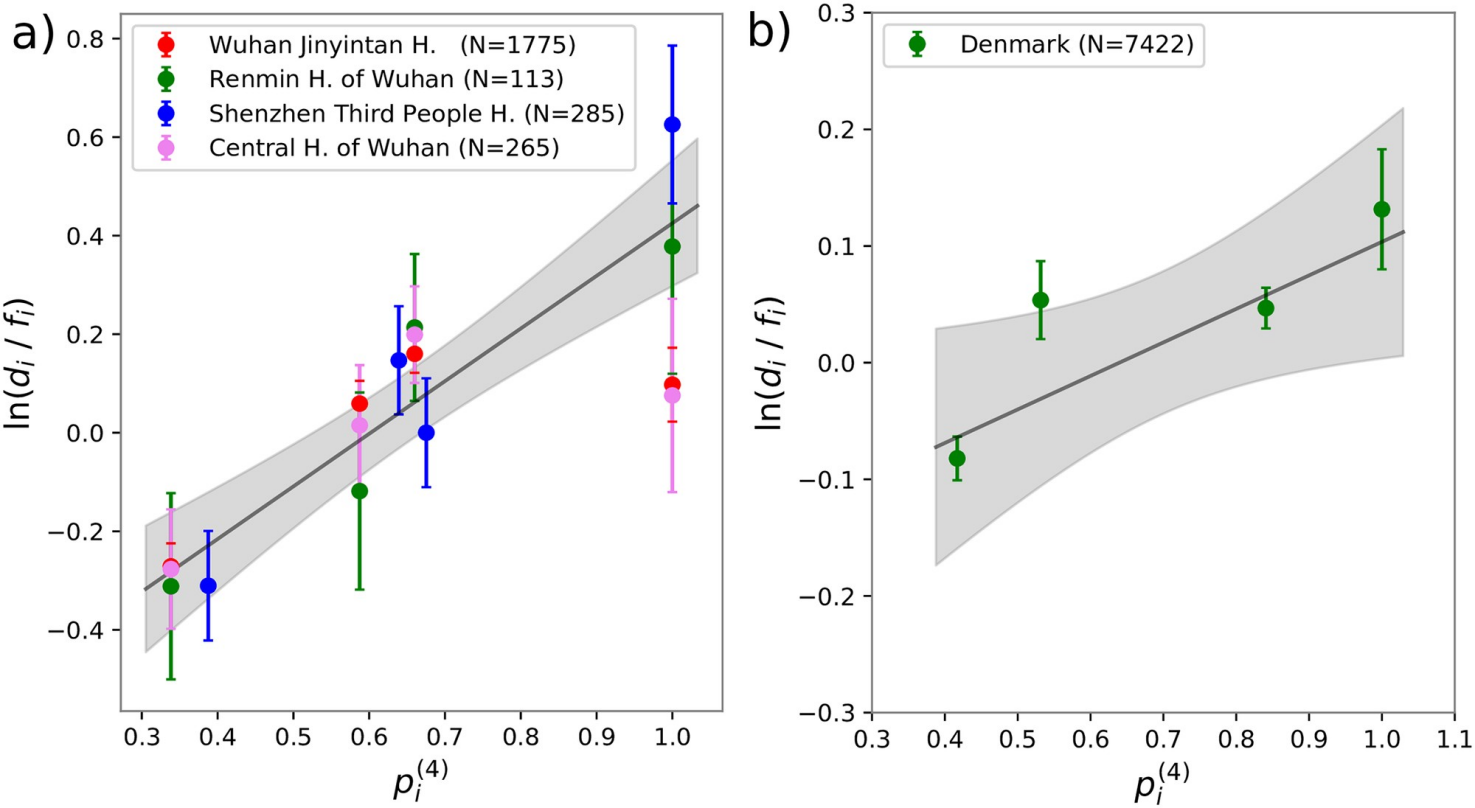
a) Ratio between frequencies of infected people for each ABO group, *d*_*i*_ over frequency of the group on the whole population, *f*_*i*_ as measured in four hospitals of the Wuhan/Shenzhen region versus its theoretical prediction, $p_{i}^{\left(4\right)}$ obtained from Eq (8) using the ABO set of rules and the blood type frequencies in Table 1. The black line represents the best solution of a linear fit. **b)** Ratio between frequencies of infected people for each ABO groups, *d*_*i*_ over frequency of the group on the whole population, *f*_*i*_ as measured in Denmark versus its theoretical prediction, $p_{i}^{\left(4\right)}$ obtained from Eq (8) using the ABO set of rules and the blood type frequencies in Table 1. The black line represents the best solution of a linear fit. The error bars on ln(*d*_*i*_/*f*_*i*_) are one standard deviation calculated by assuming a Poissonian distribution for the number of cases, thus $\Delta \left(ln\left(d_{i}/f_{i}\right)\right)=1/\sqrt{n_{i}}$ being *n*_*i*_ the number of infected people with blood type *i*.

As discussed before, the model predicts a linear relation between ln(*d*_*i*_/*f*_*i*_) and $p_{i}^{\left(4\right)}$. In (Fig 2a) we report a plot of these two quantities. The different colors represent the different datasets. The black line is the result of the least square fitting to all the data. Finally, the light grey indicates the ± one standard deviation area.

The Pearson correlation coefficient for the N = 16 data points is 0.82. The corresponding p-value (10^−4^) gives us confidence that the model is compatible with the observations.

Further data, with higher statistics, was collected by [24], based on Danish individuals found positive to COVID-19 (see Table 1). Fig 2b shows once again the logarithm of the ratio between frequencies of infected people for each ABO group, *d*_*i*_ over frequency of the group on the healthy population, *f*_*i*_ against the expected susceptibility, $p_{i}^{\left(4\right)}$. Even in this case, a correlated trend is appreciable. The Pearson correlation coefficient is 0.85 when considering all four blood groups (N = 4, p-value 0.15). If we do not consider B group data, the only one that is not statistically significant (see [24]), the correlation increases to 0.99 (N = 3, p-value 0.08). Combining the Wuhan/Shenzen datasets with the Denmark one, we obtained an overall correlation of 0.78 (N = 20, p-value <10^−4^).

To our knowledge, the five datasets, considered so far, are the only available ones for which (i) not only severely ill patients were considered and (ii) the control population was not composed of hospital patients. This allows us to test the contribution of the blood type to the sole infection transmission.

**Fig 3:**
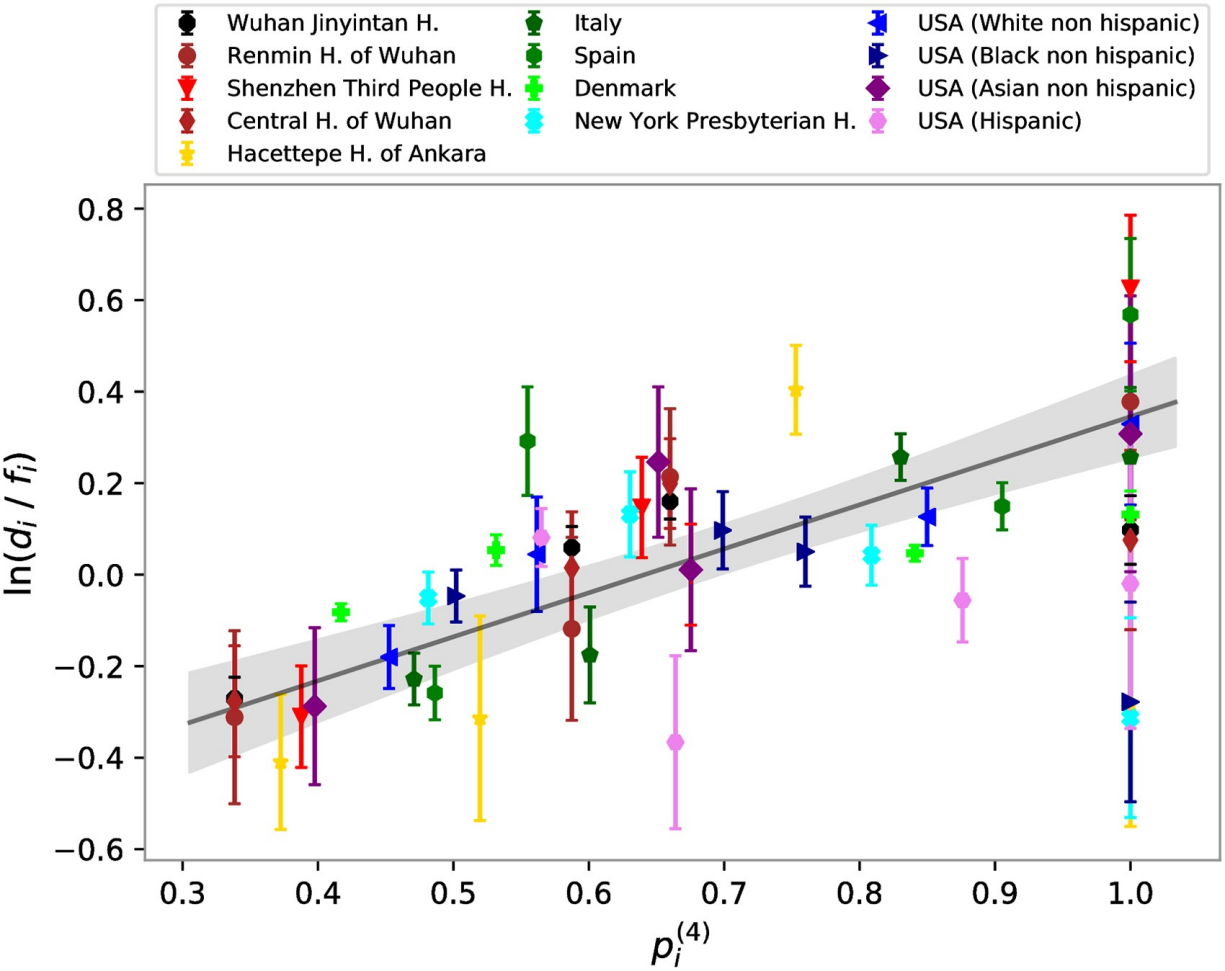
Ratio between frequencies of infected people for each ABO, *d*_*i*_ over frequency of the group on the whole population, *f*_*i*_ versus its theoretical prediction, $p_{i}^{\left(4\right)}$ obtained from Eq (8) using the ABO set of rules and all the blood type frequencies in Table 1. Black line represents the best solution of a linear fit. The error bars on ln(*d*_*i*_/*f*_*i*_) are one standard deviation calculated by assuming a Poissonian distribution for the number of cases, thus $\Delta \left(ln\left(d_{i}/f_{i}\right)\right)=1/\sqrt{n_{i}}$ being *n*_*i*_ the number of infected people with blood type *i*.

Next, we took into consideration all datasets in Table 1. In Fig 3, the logarithm of the ratio between the number of infected for each group over the group frequency of the local population (ln(*d*_*i*_/*f*_*i*_)) is compared with the 4-group susceptibility, $p_{i}^{\left(4\right)}$ computed using the data in Table 1. We observe a linear correlation of 0.53 with a p-value smaller than 10^−4^.

### Cases where infection data are not stratified by blood type

To our knowledge, all the studies reporting the blood type stratification of the infection data are reported in Table 1. However, our model allows us to analyze also not stratified infection data for which the geographical blood type distribution is known. In fact, considering the initial stages of the infection, we found that the total number of infected individuals grows as

$$\begin{array}{c} y_{T}=\sum_{i}^{k}y_{i}∼\exp\left(mt\right)\;\text{with}\;m=\beta p_{T}^{\left(k\right)}-\gamma \end{array}$$ (13)

where $p_{T}^{\left(k\right)}=\sum_{i}\sum_{j}f_{i}W_{ij}f_{j}$ (see Methods). The data of the contagion (*y*_*T*_[*t*]) by country is taken from World Health Organization (WHO) Coronavirus Disease (COVID-19) Dashboard on date 12th of June 2020 [30]. To ensure statistical reliability, we selected only countries that had registered at least 2000 positive cases from the start of the epidemic.

The incidence of the different blood types in different nations can be found in Wikipedia [31], where a collection of data and the original sources references are reported. These frequencies *f* are also listed in Table 2, together with the country ISO code. From these data we can calculate $p_{T}^{\left(2\right)}$ (keeping into account the RhD± type) and $p_{T}^{\left(4\right)}$ (keeping into account only the ABO type) for each country. Since we had to know both the infection curve data and the blood group frequencies, we ended up with 78 countries, whose information is reported in Table 2. Finally, from each curve, we extracted the effective value of the growth rate of the exponential phase, *m* with its statistical error Δ*m* as derived from the fit (see in Table 2). Additional information on data analysis is reported in the S1 File.

#### Europe and Asia

From our infection model, we expect a linear relationship between the infection rate, *m* extracted from the countries infection curves and our $p_{T}^{\left(k\right)}$. In particular, from a plot of *m* vs. $p_{T}^{\left(k\right)}$, we can both check the validity of the model and extract the parameters *β* and *γ* from which we can provide an estimate of $R_{0}=\frac{\beta }{\gamma }$.

**Fig 4:**
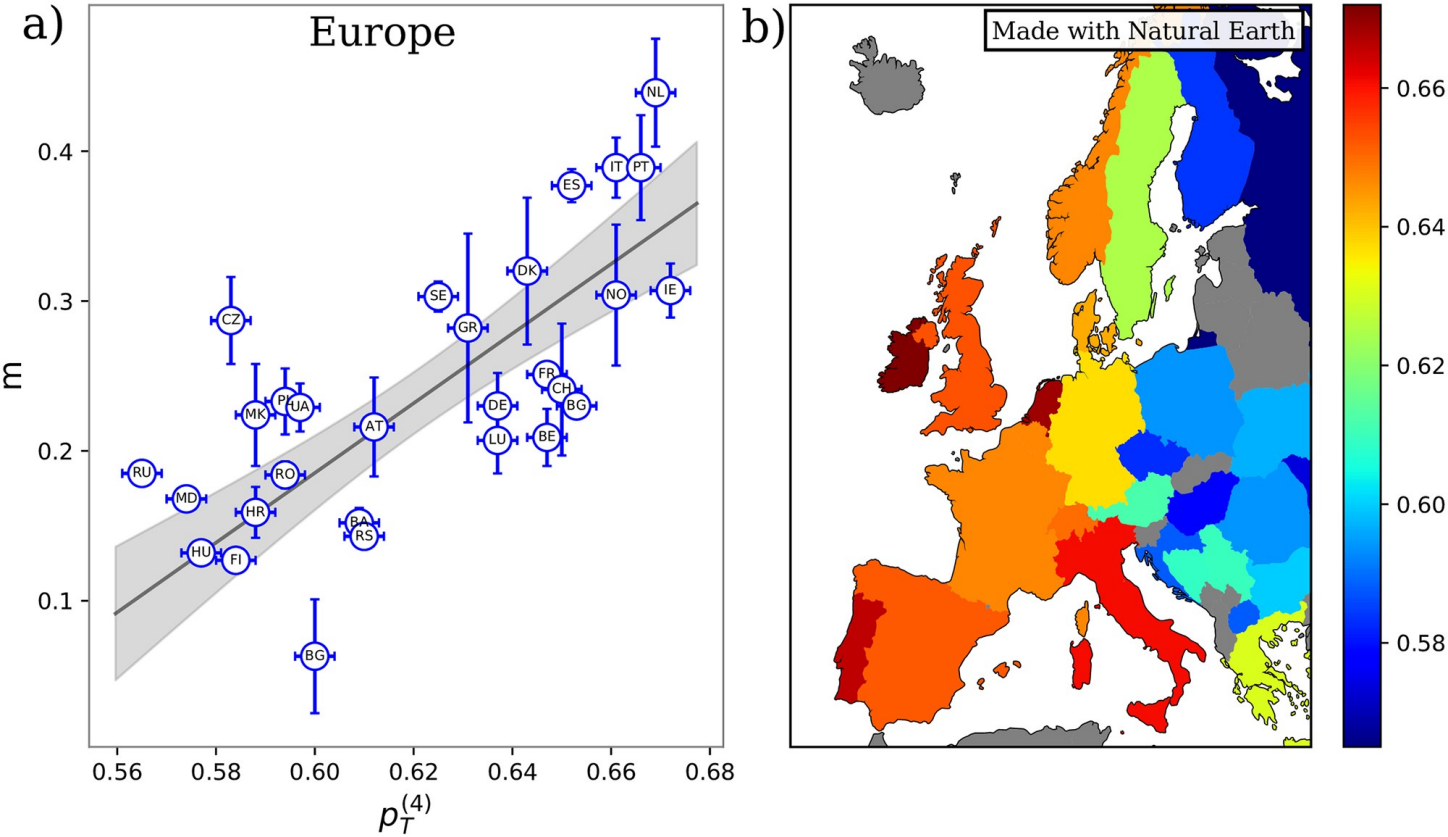
**a)** Inverse characteristic time of the epidemic exponential phase extracted from cumulative infection curves, *m*, vs theoretical prediction, $p_{T}^{\left(4\right)}$ obtained from Eq (11) using the ABO set of rules and the blood type frequencies in Table 2. Each dot corresponds to one of the 29 analyzed countries in the European region named according to the 2-letter ISO code and reported in Table 2. The black line represents the best solution of a linear fit performed with the York method and the grey shaded area is the ± one standard deviation confidence band. The uncertainty on $p_{T}^{\left(4\right)}$ are obtained as $\Delta p_{T}^{\left(4\right)}\approx 2\sqrt{4}\Delta f$ where Δ*f* = 10^−3^. **b)** Map representation of European countries. Each country is colored according to its $p_{T}^{\left(4\right)}$ susceptibility value obtained from Eq (11) using the ABO set of rules and the blood type frequencies reported in Table 2. $p_{T}^{\left(4\right)}$ values increase going from blue to red. Gray countries have not been considered due to a lack of either blood or infection information. The map shows large variability in the susceptibility $p_{T}^{\left(4\right)}$ (that ranges from 0.56 to 0.68) and a clear east-to-west gradient. The increase of susceptibility going west is a direct consequence of the tendency of increase of the O blood type in this direction: the more one blood type dominates, the higher is the susceptibility. The map has been realized using the python ‘Cartopy’ library [32] with Natural Earth data.

At first, we compared the *m* found for the 29 countries of the European area with the corresponding $p_{T}^{\left(k\right)}\text{s}$ computed starting from the frequencies of the different blood types found in each country and reported in Table 2. While a contagion scheme based only on the Rhesus group rules ($p_{T}^{\left(2\right)}$) does not explain the observed trends of the epidemics, a high Pearson correlation (0.71) is present between data and model predictions when contagions are driven by the ABO group rules ($p_{T}^{\left(4\right)}$). Indeed, as shown in Fig 4a, countries with higher susceptibility also present, on average, a higher *m* value. A linear fit of the data allows us to extract the overall values of infection (*β*) and recovery (*γ*) rates that if combined yield a value of *R*_0_ of ∼2 in excellent agreement with the current estimates of reproduction number in the early stages of the outbreak [33]. We note that points tend to form two clusters. Interestingly, retrieving the geographic information, we see a clear east-to-west gradient of the susceptibility, which drives back to the different geographical distribution of the ABH phenotypes [34]. Fig 4b clearly shows at a glance this trend for the four-group susceptibility, $p_{T}^{\left(4\right)}$.

To test the obtained linear trends, we performed an F-test assuming a constant slope as null-hypothesis. In Table 3, p-values for both the Pearson coefficient and F-statistic are reported. Note that the significance of $p_{T}^{\left(2\right)}$ is just an artifact of the data disposition, which tends to cluster around the values of $p_{T}^{\left(2\right)}=0.87$, thus yielding a high slope. Reversing the axis and repeating the linear fit, one obtains a value of F of 1.28 which has a p-value above the threshold.

**Table 3:** Main results for the different countries aggregation.

Continent		*β*	*γ*	*ρ*	Pearson	p-value	F	p-value
Europe (EU)	$p_{T}^{\left(2\right)}$	-35.39	-31.01	-	-0.26	0.17	22.4	<10^−4^
(29 countries)	$p_{T}^{\left(4\right)}$	2.31	1.20	0.52	0.71	<10^−4^	42.5	<10^−4^
Asia (AS)	$p_{T}^{\left(2\right)}$	2.06	1.80	-	0.54	0.01	19.5	<10^−3^
(21 countries)	$p_{T}^{\left(4\right)}$	4.42	2.38	0.54	0.45	0.04	1.3	0.05
South America (SA)	$p_{T}^{\left(2\right)}$	-0.46	-0.62	-	0.29	0.34	0.79	0.39
(13 countries)	$p_{T}^{\left(4\right)}$	1.24	0.67	0.54	0.62	0.02	8.1	0.02
Africa (AF)	$p_{T}^{\left(2\right)}$	0.37	0.25	0.74	-0.09	0.78	0.5	0.5
(12 countries)	$p_{T}^{\left(4\right)}$	0.52	0.22	0.42	-0.07	0.82	1.2	0.3

**Fig 5:**
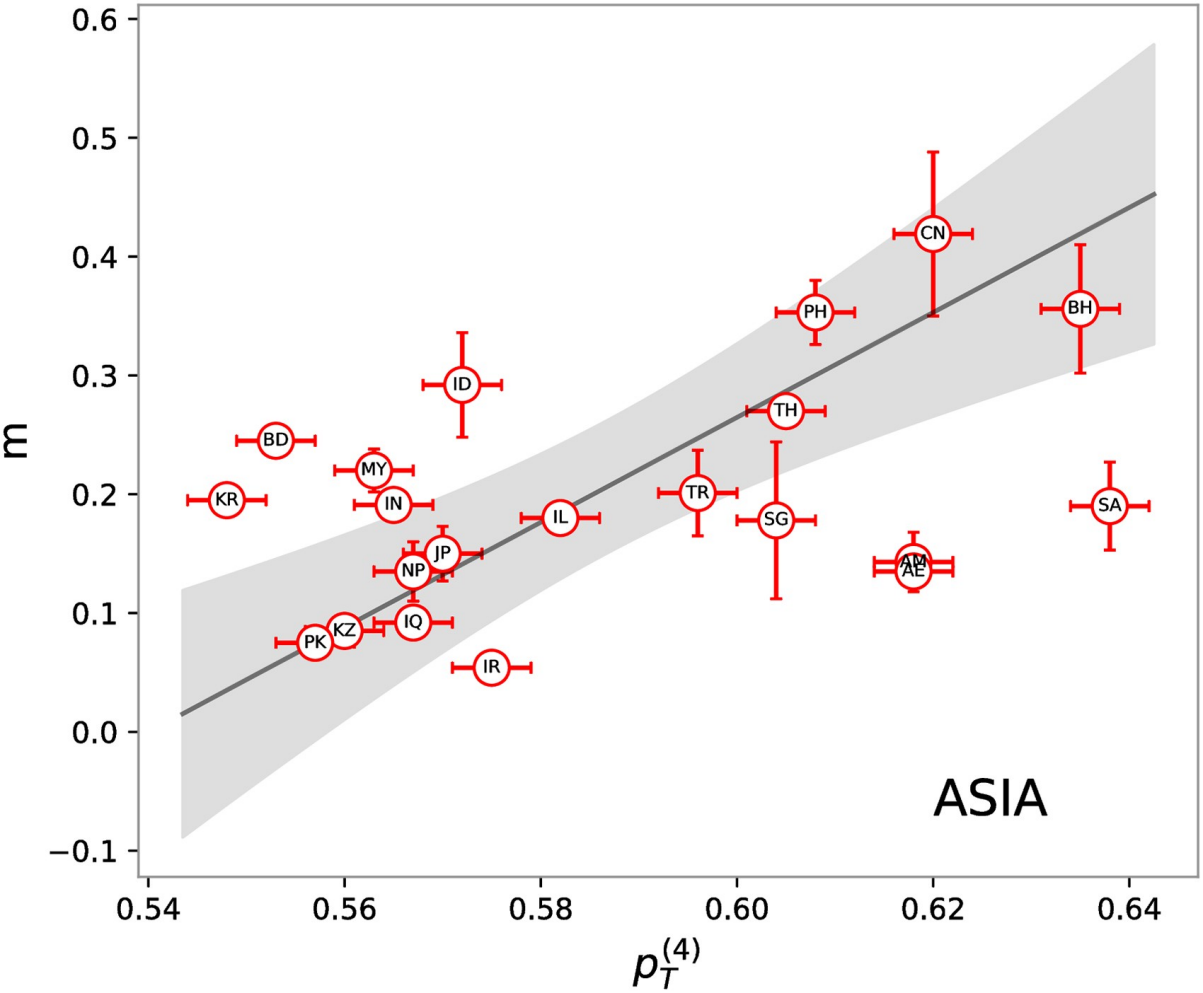
Inverse characteristic times of the epidemic exponential phase extracted from cumulative infection curves, *m* vs theoretical prediction, $p_{T}^{\left(4\right)}$ obtained from Eq (11) using the ABO sets of rules and using the blood type frequencies in Table 2. Each dot corresponds to one of the 21 analyzed countries in the Asiatic region named according to the 2-letter ISO code and reported in Table 2. The black line represents the best solution of a linear fit. The uncertainty on $p_{T}^{\left(4\right)}$ are obtained as $\Delta p_{T}^{\left(4\right)}\approx 2\sqrt{4}\Delta f$ where Δ*f* = 10^−3^.

Repeating the same analyses carried out for European countries with those in the Asia region, we found an overall similar trend (see Fig 5). In particular, $p_{T}^{\left(4\right)}$ values present a non-random linear correlation with the inverse characteristic times, *m*, of 0.45. The corresponding p-values are below the threshold of 0.05 for the 21 Asiatic countries. Testing the linear fits with the F-test, we can reject the null hypothesis with the usual threshold level of 0.05. All results are again summarized in Table 3.

#### South America and Africa

Finally, we consider two other distinct regions, i.e. South America and Africa, both characterized by a still exponentially proliferating infection. The former exhibits a trend similar to the European one, characterized by an unsubstantial contribution of the Rhesus group rules, while a correlation of 0.62 is present between the *m* coming from the fitting of the infection curves and $p_{T}^{\left(4\right)}$. The infection and recovering rates obtained by a linear fit are both smaller than those found in the Eurasian region. However, they combine to give a value of *R*_0_ of ∼2. The p-value of both the Pearson correlation and the F-test on the linear fit is below the significance threshold of 0.05 for all sets of rules except for $p_{T}^{\left(2\right)}$ one. Results for the African region instead show no meaningful correlation. As it is the slope of the best linear fit which does not pass an F-test with zero slope as a null hypothesis.

#### Final comments on the countries cases

Overall, we found a statistically significant correlation among the *m* and $p_{T}^{\left(4\right)}$ data for Europe, Asia, and South Americas, taken individually. The reason for analyzing separately these continents lies in the fact that we expect that besides blood type distribution other factors affect the infectivity onset and initial growth rate. The lifestyle and the local climate are certainly some of them. We have therefore considered countries aggregations that preserve at the best these two aspects. To check this hypothesis, we have analyzed (see Table 4) what is the effect of adding North America and/or Australia to the European countries. As can be seen in Table 4, the Pearson correlation does not change significantly in these cases. A rather worst result is obtained by considering Asia and Europe together, although the Pearson correlation still maintains a high degree of statistical significance (p-value better than 10^−4^). The correlation becomes even worst when considering the whole world (Pearson 0.43) but also in this case there is a great advantage (p-value better than 10^−3^) with respect to the null hypothesis (no-correlation between *m* and $p_{T}^{\left(k\right)}$). We conclude that the model presented here is compatible with the existing data.

**Table 4:** Pearson correlations between *m* and $p_{T}^{\left(4\right)}$ for different continent aggregations.

Aggregation	Countries	Pearson	p-value
Europe and North America	31	0.70	<10^−4^
Europe and Australia	30	0.69	<10^−4^
Europe and Asia	50	0.62	<10^−4^
Temperate (North)	48	0.63	<10^−4^
Tropical	26	0.24	0.24
Temperate (Sud)	4	0.97	0.03
World	78	0.43	<10^−3^

As far as the effect of temperature, humidity, etc. it is tempting to speculate on the obtained results: *i)* Europe and Asia share a good correlation, as well as the values of *β* and *γ*; *ii)* South America has again a good correlation, but its *β* and *γ* are smaller than in Europe and Asia; *iii)* Africa shows a bad correlation. These three points could be rationalized remembering that, at the pandemic initial stage Europe and Asia were in their wintertime, South America in the summertime, while African countries experienced different climate situations.

## Discussion

Most of the proteins that decorate cell membranes are bound to glycans [35]. The presence of those carbohydrate chains provides a further channel of interaction between proteins, besides the usual direct protein-protein one, and evolved to play a large array of life-sustaining functions including support, signaling, protein folding, and protection.

It has been suggested that protection against pathogens was the driving force that favored the evolution of the complex landscape of glycan interactions (see e.g. [36] for a more detailed discussion). Since viruses do not have genes for glycan synthesis or modification, they inherit host cell glycans after each round of replication in a new host. This means that the host cell in which the virus last replicated generated the glycans on viral glycoproteins [36].

A mutation in the host population, having as an outcome the loss of a glycan modification, could then provide a selective advantage to the glycan-lacking subpopulation. In fact, pathogens using that glycan as a receptor would not be able to invade the host cells anymore. Moreover, the host can develop specific antibodies against the abolished glycan [37]. Human blood groups constitute an important example. Individuals having O blood group lack A or B antigens and when presented with glycan motifs similar to either A or B antigens, they develop anti-A and anti-B antibodies. Individuals with A or B blood group develop either anti-B or anti-A antibodies, respectively. On the other hand, people with subgroup AB are not able to develop such antibodies. According to those “rules”, in case of an infection, one would expect that looking at the blood type of the people found infected by the virus, individuals with group O should be under-represented with respect to their occurrence in the whole population. This feature has been indeed observed by Zhao and coworkers [21] for the COVID-19 outbreak, caused by the novel SARS-CoV-2 coronavirus. Notably, a similar behavior was found in the hospital outbreak of SARS in Hong Kong in 2003 [38] and in that of the West Nile virus in Greece in 2010 [39]. Moreover, both SARS-CoV and SARS-CoV-2 S protein trimers are covered by an extensive glycan shield, surrounding the receptor-binding domain and can either infect or not infect cells that express ABH antigens depending on the specific individual phenotype [40, 41]. Importantly, these hypothesized asymmetrical transmission rules should affect the number of people with a certain blood type that can be potentially be infected because they do not allow all infected to infect any potentially contacted person. Consequently, we have a dilution effect on the contagion that must be reflected in the growth of the epidemics. In the present work, we have studied how the existence of asymmetrical virus transmission rules affects such growth, and how the fraction of infected patients with a certain blood type should not depend only on the patient blood type but on the number of infected people that may infect him.

To this aim, we expanded the usual SIR formalism to take into consideration the possible effect of blood antigenicity in the COVID-19 transmission and provided analytical solutions in the small-time limit, where the epidemic is in its exponential-growing regime. We obtained an expression linking the inverse characteristic time of the exponential phase with the abundances of the different blood groups in the population. We propose a set of susceptibility indices: $p_{i}^{\left(k\right)}$ for the sub-population having blood group *i* and $p_{T}^{\left(k\right)}$ for the total population (where *k* are the number of the considered blood types).

The model predicts a linear dependence for both the logarithm of the ratio $\frac{d_{i}}{f_{i}}$ and the observed epidemic inverse timescale *m* when compared against the susceptibilities. To test the model, we first compare its predictions with the experimental data provided in [21, 29], where the blood type of infected people of two Chinese regions was collected. Comparing the population frequencies of blood groups with those found in the infected sub-population, we verified that the proposed contagion scheme well describes the observed frequencies, since the difference observed between the ABO blood type population distribution and the ABO blood type infected people distribution supports the validity of the infection scheme proposed in [23]. A similar approach has been used to test the model for the Denmark data [24], characterized by overall higher statistics, and again we found good accordance with the model.

Next, we consider all data reported in Table 1. Notably, the linear trend remains although data points are more scattered. We noted that most of the added sets of data were obtained considering only severely ill individuals and hospitalized individuals as a negative control. While our model only accounts for the effects of blood groups in the transmission of the virus, the ABH phenotype could in principle impact both the course of the COVID disease and the general health of individuals. Consequently, considering severely ill patients and hospitalized people as controls may introduce biases in both the *d*_*i*_ and *f*_*i*_ distributions, which reflect on a higher dispersion of the data in Fig 3.

To further validate our model, we analyzed the infection curves of a large set of countries worldwide, comparing the characteristic time of the infection outbreak with the prediction of our model based on the known blood group distribution for each country. Note that in this second case, we do not have the information about the blood groups of the infected population but only on the whole population. Thus, we can compare the growth rate in the short time, exponential, phase with the “susceptibility” $p_{T}^{\left(k\right)}$ proposed by the model.

We managed to collect data on the blood frequencies and the infections for 78 different countries belonging to four well distinct geographical areas, i.e. Europe, Asia, South America, and Africa. Since we expect that differences both geographical and on lifestyles affect the infection and recovering rates, we kept the countries separated according to the four identified areas. This allows us to discard possible bias due to data collection and geographical factors. Using a set of rules based on Rhesus blood types, we observed an always not significant correlation. From the point of view of the biological interpretation of the results, this is not surprising. A set of rules solely based on the RhD factor would not support the working hypothesis, which is based on the presence of glycan antigens on the viral capsid. This could be because RhD antigens are proteins and so can not be easily inherited by the virus as ABO group antigens, which are glycans. Moreover, RhD- individuals do not have innate antibodies without exposure to RhD+ blood.

On the contrary, considering the ABO set of rules, three out of the four areas present a very good agreement between data and model prediction (see Table 3). Summing up, it seems that the ABO blood type has an effect on the virus spreading pattern.

## Conclusion

In a nutshell, we proposed a generalized SIR model with infection rules dictated by antigenicity between different blood types. Obtaining an analytical solution of the model for the exponential phase, we were able to provide a rigorous theoretical test of the hypothesis proposed in [23]. We have made the test both for local data, where the number of infected people is stratified by blood type, and, on a wider scale, analyzing the infection growth curves of 78 countries worldwide. Overall, the present study concludes that the hypothesis of a blood type effect on the COVID-19 advanced in [23] it is compatible with the available observational epidemic data. Obviously, to strengthen the validation a direct detection of the antigens linked to the SARS-CoV-2 is needed, but this goes far beyond the goals of the present paper, which aims at presenting a mathematical framework to validate the hypothesis discussed in [23] on patients and countries data. Moreover, this formalism allows us to understand the relation between the regional infection growth rate and the population blood type distribution. As a final note, we observe that, besides blood types, other population-dependent antigens distribution may play a role in the geographically heterogeneous infection spreading [42].

## Supporting information

S1 File (PDF)

S1 Graphical abstract (TIF)
