# Supplementary material for: Does blood type affect the COVID-19 infection pattern?
Source: PLoS One. 2021 May 13;16(5):e0251535. doi: 10.1371/journal.pone.0251535 (PMC8118288; doi:10.1371/journal.pone.0251535)
Supplement: S1 File — (PDF) [file pone.0251535.s001.pdf]

Does blood type affect the COVID-19 infection pattern?

Mattia Miotto,[1,2] Lorenzo Di Rienzo,[2] Giorgio Gosti,[2] Edoardo Milanetti,[1,2] and Giancarlo Ruocco[2,1]

[1] *Department of Physics, University of Rome 'La Sapienza', Piazzale Aldo Moro, 5, I00185, Rome, Italy*
[2] *Fondazione Istituto Italiano di Tecnologia (IIT), Center for Life Nano & Neuroscience, Viale Regina Elena 291, I00161 Roma, Italy*

I. STANDARD AND GENERALIZED SIR MODEL

In this section we first consider the standard SIR model for the time evolution of the fraction of susceptible ($x(t)$), infected ($y(t)$), and recovered ($z(t)$) people in a population of size N, recalling the main features of the model and its solution[2].

We then analyze in detail the generalization of the SIR model to a more complex pattern of infection.

A. Standard SIR model

The standard SIR model is ruled by the following equations:

$$\frac{dx(t)}{dt} = -\beta\, x(t)\, y(t) \tag{1}$$
$$\frac{dy(t)}{dt} = \beta\, x(t)\, y(t) - \gamma\, y(t) \tag{2}$$
$$\frac{dz(t)}{dt} = \gamma\, y(t) \tag{3}$$

satisfying the normalization relation:

$$x(t) + y(t) + z(t) = 1 \tag{4}$$

and β and γ are parameters representing the infection and recovery rate respectively.

By defining $\rho \doteq \gamma/\beta$ and re-scaled time $\tau \doteq \beta t$, the equations (1) - (3) are promptly rewritten as:

$$\dot{x} = -\beta\, x\, y \tag{5}$$
$$\dot{y} = \beta\, x\, y - \gamma\, y \tag{6}$$
$$\dot{z} = \gamma\, y. \tag{7}$$

From now on, the dot indicates differentiation with respect to τ.

As initial conditions we set:

$$x(0) = x_o \tag{8}$$
$$y(0) = y_o = 1 - x_o \tag{9}$$
$$z(0) = 0. \tag{10}$$

We thus chose the simple and realistic case of a few infected people at time zero, and all the remaining population susceptible of infection.

1. *Solutions*

The first step to solve the set (5-7) is performed by eliminating y from (5) and (7):

$$\dot{z} = -\rho \frac{\dot{x}}{x} \tag{11}$$

or, by simplifying $d\tau$,

$$\frac{dz}{dx} = -\rho \frac{1}{x}, \tag{12}$$

thus

$$z(\tau) - z_o = -\rho \log(x(\tau)/x_o). \tag{13}$$

Finally, setting the arbitrary constants at $\tau = 0$ ($z_o = 0$ and x_o), we get the solution for $x(\tau)$:

$$x(\tau) = x_o \; e^{-z(\tau)/\rho} \tag{14}$$

The solution for y is obtained from (4):

$$y(\tau) = 1 - z(\tau) - x_o \; e^{z(\tau)/\rho}. \tag{15}$$

As for the solution for $z(\tau)$, we start by differentiating (7) and using (4):

$$\ddot{z} = \rho \dot{y} = \rho(-\dot{x} - \dot{z}). \tag{16}$$

Thus, rearranging

$$\ddot{z} + \rho \dot{z} = -\rho \dot{x} = \dot{z} x = \dot{z} x_o \; e^{z(\tau)/\rho} \tag{17}$$

Finally, the differential equation for $z(\tau)$:

$$\ddot{z} + \rho \dot{z} - \dot{z} x_o \; e^{z(\tau)/\rho} = 0 \tag{18}$$

is of Bernoulli type, and its solution - with the condition $z(0) = 0$ - is implicitly given by :

$$\tau = \frac{1}{\rho} \int_0^{z(\tau)} \frac{d\zeta}{1 - \zeta - x_o e^{-\zeta/\rho}}, \tag{19}$$

Recapitulating, for the standard SIR model one finds:

$$x(\tau) = x_o \; e^{-z(\tau)/\rho} \tag{20}$$
$$y(\tau) = 1 - z(\tau) - x_o \; e^{-z(\tau)/\rho} \tag{21}$$
$$\tau = \tfrac{1}{\rho} \int_0^{z(\tau)} \tfrac{d\zeta}{1-\zeta-x_o e^{-\zeta/\rho}}, \tag{22}$$

2. Infectivity maximum

The solution for $y(\tau)$ shows the usual peak of infection. The fraction of infected people at the maximum is an important parameter for the description of the pandemic evolution.
From Eq. (7), the maximum of infectivity, y_M, is found when $\ddot{z}$=0.
According to Eq. (17), imposing $\ddot{z}$=0 is equivalent to:

$$\dot{z}(\rho - x_o \; e^{z(\tau)/\rho}) = 0 \tag{23}$$

Beside the trivial solution $\dot{z} = 0$, which takes place at $\tau \to \infty$, the value of $z(\tau)$, z_M, when $y(\tau)$ attains its maximum y_M, is

$$z_M = \rho \log(x_o/\rho). \tag{24}$$

Finally, we found:

$$y_M = 1 - \rho + \rho \log(\rho). \tag{25}$$

3. Width of the infectivity peak

The values of $z(\tau)$ for which $y(\tau)$ reaches half-height values, z_- e z_+, are given by:

$$\begin{aligned} z_+ &= \frac{1}{2}(1+\rho-\rho\log(\rho)) + \rho W_o(\frac{1}{\rho} e^{\frac{-(1+\rho-\rho\log(\rho))}{2\rho}}) \\ z_- &= \frac{1}{2}(1+\rho-\rho\log(\rho)) + \rho W_{-1}(\frac{1}{\rho} e^{\frac{-(1+\rho-\rho\log(\rho))}{2\rho}}) \end{aligned}$$

Where W_o and W_{-1} are the first and second branch of the Whittaker functions.
The corresponding τ_- e τ_+ can be find using Eq. 22. It results:

$$\Delta\tau = \frac{1}{\rho} \int_{z_-}^{z_+} \frac{d\zeta}{1 - \zeta - x_o e^{-\zeta/\rho}} \tag{26}$$

Short time expansion

It is well know that the initial stage of the infection is well represented by an exponential growth. It is therefore useful to perform the expansion of the solutions (20), (21) and (22) for $\tau \to 0$.
In the small τ limit, the expression for $\tau(z)$ given by Eq. (22), becomes:

$$\tau(z) \approx \tau|_{z=0} + \frac{d\tau}{dz}\Big|_{z=0} z = \frac{1}{\rho} \frac{z}{(1 - x_o)},$$

thus

$$z(\tau) \approx \rho(1 - x_o)\tau = \rho y_o \tau. \tag{27}$$

By substituting in (20) and (21), we get

$$x(\tau) \approx x_o\, e^{-y_o\tau} \tag{28}$$
$$y(\tau) \approx 1 - \rho y_o \tau - x_o\, e^{-y_o\tau}. \tag{29}$$

The last equation, by expanding the exponential, collecting the terms linear in τ and resuming the exponential, becomes:

$$y(\tau) \approx y_o\, e^{(x_o-\rho)\tau} \tag{30}$$

which represents the desired exponential growth of the infection at short time. As at short time the fraction of infected population is very small (i.e. $y_o \ll 1$), we can safely approximate $x_o \approx 1$:

$$y(\tau) \approx y_o\, e^{(1-\rho)\tau}. \tag{31}$$

Equation (31) expresses one of the key concepts of epidemic models. The number of infectious individuals grows exponentially if $\rho < 1$, or $\beta > \gamma$. Often, in the literature, the parameter controlling the level of infection growth is the "reproduction number", R_o, defined as the average number of secondary infections caused by a primary case introduced in a fully susceptible population[1]. R_o is therefore equal to β/γ, which, in our notation, means $R_o = 1/\rho$.
From those simple considerations arises the concept of epidemic threshold: only if $(1-\rho) > 0$, thus $R_0 > 1$ (i.e. if a single infected individual generates on average more than one secondary infection), an infective agent can cause an outbreak. If $R_0 < 1$ (i.e. if a single infected individual generates less than one secondary infection), then $(1-\rho) < 0$ and the initial stage of the epidemic is characterized by a *decreasing* number of cases.

Summary of standard SIR properties

Here, we recollect the main results from previous Sections. The main results are four. First, it is worth to always perform the limit $x_o \to 1$, while keeping $y_o \neq 0$ as discussed below. This allows us to correctly capture the exponential growth that characterizes the initial phase of an epidemic.
Second,the exact solutions of the standard SIR model wit the initial conditions are:

$$\begin{aligned} x(\tau) &= e^{-z(\tau)/\rho} \\ y(\tau) &= 1 - z(\tau) - e^{z(\tau)/\rho}. \\ \tau &= \frac{1}{\rho}\int_0^{z(\tau)} \frac{d\zeta}{1-\zeta-e^{-\zeta/\rho}} \end{aligned}$$

Third, the value of maximum infection is found to be:

$$y_M = 1 - \rho + \rho\log(\rho).$$

Fourth, at short time, when the exponential growth dominate the solution, the following approximations hold:

$$\begin{aligned} x(\tau) &= e^{-y_o\tau} \\ y(\tau) &= y_o\, e^{(1-\rho)\tau} \\ z(\tau) &= \rho y_o \tau \end{aligned} \tag{32}$$

As last observation, it is worth to emphasize that Eq. (32) is identical to the short time expansion of the infectivity in the SIS (Susceptible-Infected-Susceptible) model, which is a simplified version of the SIR model where individuals never acquire immunity to the infection. The latter, at short time, reads:

$$\dot{y}(\tau) = x_o y(\tau) - \rho y(\tau) \tag{33}$$

whose solution coincides with Eq. (30)) or, after the $x_o = 1$ approximation, with Eq. (31)).

Generalized SIR model

In this section, we present a general SIR model, which describes the evolution of the epidemic assuming that transmission of the infection depends on the blood groups of the individuals. In particular, we will show how to derive proper descriptors that take into consideration not only transmission rules based on the ABO group but also possible combinations of different groups. As examples, we discuss the hypothetical cases of a two-group set of rules (i.e. the Rhesus group), the ABO group, and their combination. We note that from a biological point of view, it seems improbable that the RhD system could play a role in the infection transmission however the derived framework could be applied to other kinds of glycan-based groups, like the Lewis one.
As we said, our aim is to generalize the SIR model to the case where the population is not homogeneous, but it is composed of different sub-populations that follow specific infection rules.
Let us first discuss in detail the significant case in which there are only two diverse sub-populations.

4. Two sub-populations

Let us discuss the simple case where two subpopulation (1 and 2) exists, and the rules are such that people belonging to sub-population 1 can be infected only by themselves, while people of subpopulation 2 can be infected by both sub-groups 1 and 2.

In this case, the SIR equations for the variables $x_i(\tau)$, $y_i(\tau)$ and $z_i(\tau)$, with i=1,2, read:

$$\dot{x}_1 = -x_1\, y_1 \tag{34}$$
$$\dot{y}_1 = x_1\, y_1 - \rho\, y_1 \tag{35}$$
$$\dot{z}_1 = \rho\, y_{\cdot 1} \tag{36}$$
$$\dot{x}_2 = -x_2\, (y_1 + y_2) \tag{37}$$
$$\dot{y}_2 = x_2\, (y_1 + y_2) - \rho\, y_2 \tag{38}$$
$$\dot{z}_2 = \rho\, y_2 \tag{39}$$

Here we assume that the parameters β and γ are the same for all the population. The fraction of people belonging to the i-th sub-population is f_i. As intuitively, the following relations holds: $f_1 + f_2 = 1$, $0 \leq x_i(t\tau) \leq f_i$, $0 \leq y_i(t\tau) \leq f_i$, $0 \leq z_i(t\tau) \leq f_i$, and $x_(\tau) + y_(\tau) + z_i(\tau) = f_i$.

Similarly to the general case, we chose as initial conditions:

$$x_1(0) = x_1^o = f_1 x_o \tag{40}$$
$$y_1(0) = y_1^o = f_1 y_0 = f_1(1 - x_o) \tag{41}$$
$$z_1(0) = 0 \tag{42}$$
$$x_2(0) = x_2^o = f_2 x_o \tag{43}$$
$$y_2(0) = y_2^o = f_2 y_0 = f_2(1 - x_o) \tag{44}$$
$$z_2(0) = 0 \tag{45}$$

To our knowledge the solution to the set of equations (34 - 39) is only numerical. An example of the solution, for the case $f_1 = 0.4$, $f_2 = 0.6$ and $\rho = 0.1$, is reported in Figure 1. Here only $y(\tau)$ is shown: the red and green dashed lines represent the quantity $y_1(\tau)$ and $y_2(\tau)$ respectively, while the full blue line is the total number of infected $y(\tau) = y_1(\tau) + y_2(\tau)$.

The horizontal dotted line indicates the maximum value that would have been reached by $y(\tau)$ if the case of all-infect-all rule (standard SIR model).

Obviously, the number of infected is lower in this case, as the sub-population 2 cannot infect person of sub-population 1, thus reducing the "effective" infection rate. However, the time evolution of the infection is not a simple re-scaling of the β parameter, the evolution for $y_1(\tau)$ and $y_2(\tau)$ show different maxima at different times, so to produce a more rich scenario.

For a given ρ value, the maximum of infection depends on the distribution of the two sub-populations. Clearly for $f_1 = 0$ or $f_1 = 1$, the standard SIR situation is recovered, while for $f_1 = f_2 = 0.5$ this maximum attains its minimum value. A plot of y_M as a function of f_1/f_2 is reported in Figure 1 of the Main Text.

Although an analytic solution to the set of equations (34 - 39) is not known, we can perform a short time expansion. As discussed in the standard SIR case, the short time expansion for the variable(s) $y_i(\tau)$ involve only the variables themselves:

$$\dot{y}_1 = x_1^o\, y_1 - \rho\, y_1 \tag{46}$$
$$\dot{y}_2 = x_2^o\, (y_1 + y_2) - \rho\, y_2 \tag{47}$$

Searching solution of the type:

$$y_i(\tau) = y_i^o\, e^{\pi_i \tau} \tag{48}$$

and using the initial conditions (40 - 45) we get:

$$\pi_1 = f_1 - \rho \tag{49}$$
$$\pi_2 = 1 - \rho \tag{50}$$

The short time expansion thus reads:

$$y_1(\tau) \;=\; y_o f_1 \, e^{(f_1-\rho)\tau} \tag{51}$$
$$y_2(\tau) \;=\; y_o f_2 \, e^{(1-\rho)\tau} \tag{52}$$

Also important is the total number of infected people, not stratified for the specific sub-population, $y(\tau) = y_1(\tau) + y_2(\tau)$. This quantity, in the short time limit, is promptly calculated from Eqs. (51 - 52) as:

$$y(\tau) = y^o \, e^{\pi_T \tau} \tag{53}$$

with

$$\pi_T = f_1^2 + f_2^2 + f_1 f_2 - \rho \tag{54}$$

B. General case of k sub-population

In the general case of k sub-populations $i = 1, ..., k$, the time evolution of the variables can be written as:

$$\dot{x}_i \;= -x_i \sum_{j=1}^{k} W_{ij} y_j \tag{55}$$
$$\dot{y}_i \;= x_i \sum_{j=1}^{k} W_{ij} y_j - \rho \, y_i \tag{56}$$
$$\dot{z}_i \;= \rho \, y_i \tag{57}$$

where the matrix $\mathbf{W}$ encodes the infection rules.

As first example, we discuss the simple case of only two sub-populations that could follow the infection rules associated to the RhD$\pm$ blood type ($f_1 \equiv f_-$, $f_2 \equiv f_+$, where $f_\pm$ is the frequency of one blood type in the population). We assume the the sub-population with RhD+ cannot infect the RhD$-$ one. On the other hand, the RhD$-$ sub-population can infect both RhD$-$ and RhD+ sub-populations. The matrix $\mathbf{W^{(2)}}$ in this situation turns out to be:

$$\mathbf{W^{(2)}} = \begin{pmatrix} 1 & 0 \\ 1 & 1 \end{pmatrix} \tag{58}$$

The corresponding SIR equations, to our knowledge, cannot be solved by quadrature. This example is discussed in some details in Supporting Information, where it is also reported the short time expansion. The numerical solution for this $k = 2$ case is also depicted in Figure 1, where as an example the fraction of infected people $y_i(\tau)$ for the two sub-populations (green dashed and red dotted lines) and the total fraction $y_T(\tau)$ of infected people (blue line) are reported as a function of the reduced time τ for the case $\rho = 0.1$ and $f_1 = 0.4$, $f_2 = 0.6$.

One can immediately notice that the maximum fraction of infected people, y_M, is reduced with respect to the one-population case (horizontal dash-dotted blue line). This is a trivial consequence of the impossibility of '+' to infect '-', thus reducing the "effective" infection rate. The dependence of the maximum infectivity on f_1 ($f_2 = 1 - f_1$) is reported in the inset of Figure 1 for three representative values of ρ (ρ=0.1, 0.3 and 0.5). Noteworthy, for the case ρ=0.5, i.e. R_0=2, the actual value observed in Europe, the maximum of infectivity is reduced from the all-infect-all case ($\approx$0.2) to a value more than ten times smaller ($\approx$0.01) when f_1 approach 0.5. The immediate consequence of this finding is that the time evolution of the epidemic strongly depends on the blood type distribution, giving a qualitative explanation of the observed high geographical variability: even a small change in f_1/f_2 highly affects both the infectivity maximum and the infectivity growth rate in the initial exponential growth phase.

If we turn to consider the more biologically relevant case of the ABO types, we have four subpopulations with the corresponding frequencies: $f_1 \equiv f_0$, $f_2 \equiv f_A$, $f_3 \equiv f_B$, $f_4 \equiv f_{AB}$). With the infection rules summarised in Figure **??** the $\mathbf{W^{(4)}}$ matrix results:

$$\mathbf{W^{(4)}} = \begin{pmatrix} \mathbf{W^{(2)}} & \mathbf{0} \\ \mathbf{W^{(2)}} & \mathbf{W^{(2)}} \end{pmatrix} \tag{59}$$

Finally, assuming that both the ABO and the RhD types would play a role, there are eight subpopulations with respective frequencies $f_{O-} \equiv f_1$, $f_{A-} \equiv f_2$, $f_{B-} \equiv f_3$, $f_{AB-} \equiv f_4$, $f_{O+} \equiv f_5$, $f_{A+} \equiv f_6$, $f_{B+} \equiv f_7$, $f_{AB+} \equiv f_8$, and

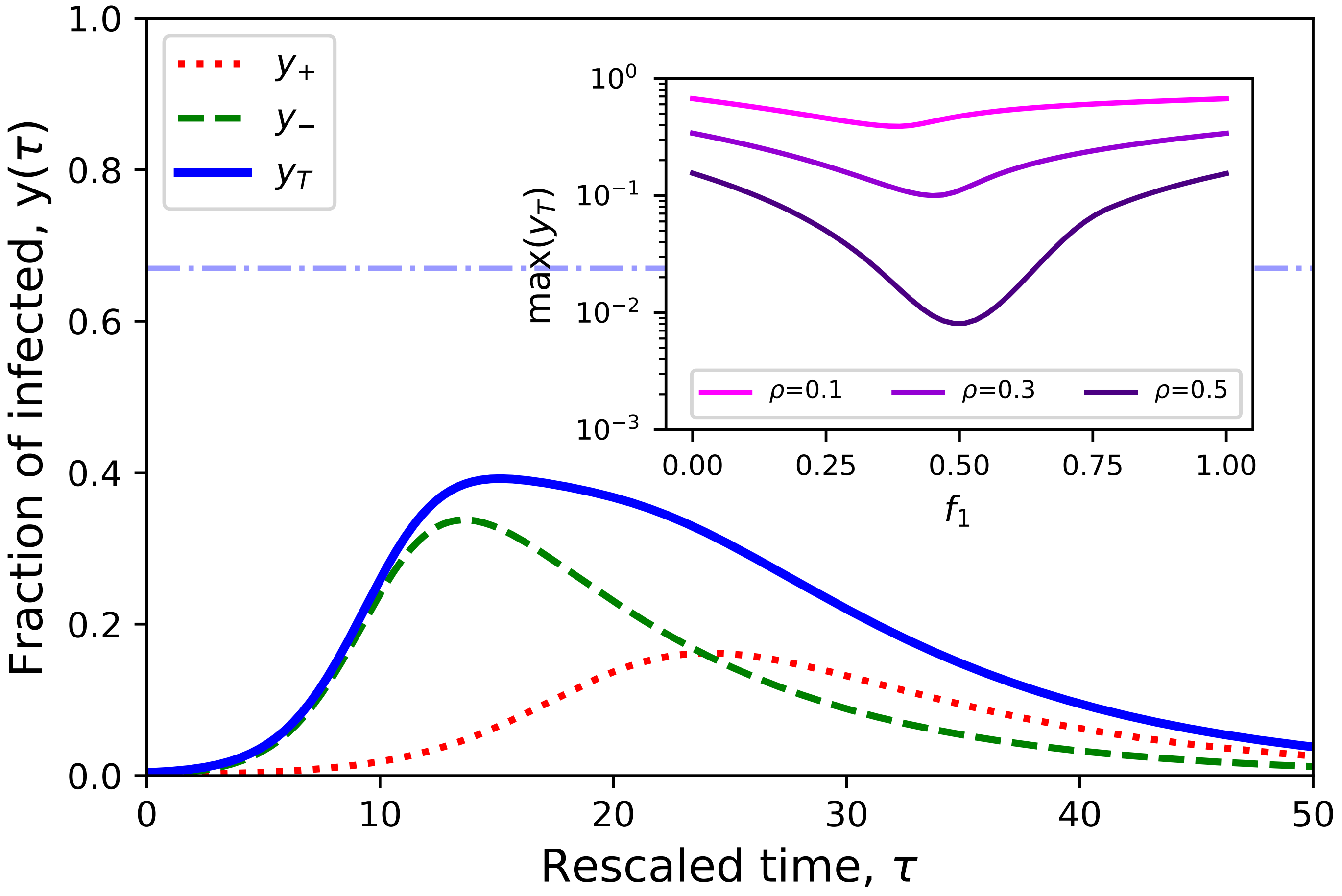

FIG. 1: Example of numerical solution of the generalised SIR model for two sub-populations ($k = 2$) and for the infectivity matrix of Eq. (58). The reported example is for $\rho = 0.1$, $f_1 = 0.4$ and $f_2 = 0.6$. The dashed and red lines represent the fraction of infected people in the two sub-populations, the blue line the total fraction of infected people, the horizontal dotted line indicates the maximum value, y_M, of $y_T(\tau)$ in the case of all-infect-all rule. The inset report the maximum of $y_T(\tau)$ as a function of f_1 ($f_2 = 1 - f_1$) for three distinct values of ρ.

the corresponding matrix $\mathbf{W^{(8)}}$ reads:

$$\mathbf{W^{(8)}} = \begin{pmatrix} \mathbf{W^{(4)}} & \mathbf{0} \\ \mathbf{W^{(4)}} & \mathbf{W^{(4)}} \end{pmatrix} \tag{60}$$

The recursive structure of Eqs. (58 - 60) has a simple explanation, that can be better understood if we think of the ABO blood type system as the combination of two codominant and one recessive allele that form two independent systems. Let's specify if a person does have ("+") or does not have ("−") the antigen A (A+ or A− respectively), analogously for the antigen B (B+ or B−). Then the usual blood type is: 0 ≡ [A−,B−]; A ≡ [A+,B−]; B ≡ [A−,B+]; AB ≡ [A+,B+]. With this notation it is clear that the "ABO" system (which follows $W^{(4)}$) is the product of the "A" system (which follows $W^{(2)}$) and the "B" system (which again follows $W^{(2)}$): ABO=A± × B±. Furthermore, by multiplying the ABO system by the RhD± system we get the ABO × RhD± = A± × B± × RhD± system, which obeys the $W^{(8)}$ infection rules. Summing up, any time it exists a set of antigens, A_i, $i = 1..a$, that can be either present or absent, the infection rule of the $A_1 \times A_2 \times .... \times A_a$ system follows the $W^{(k)}$ infection rule with $k = 2^a$.

The set of $3k$ differential equations (55 - 57), together with the initial conditions:

$$\begin{aligned} x_i(0) &= f_i x_o \approx f_i \\ y_i(0) &= f_i y_o \\ z_i(0) &= 0 \end{aligned} \tag{61}$$

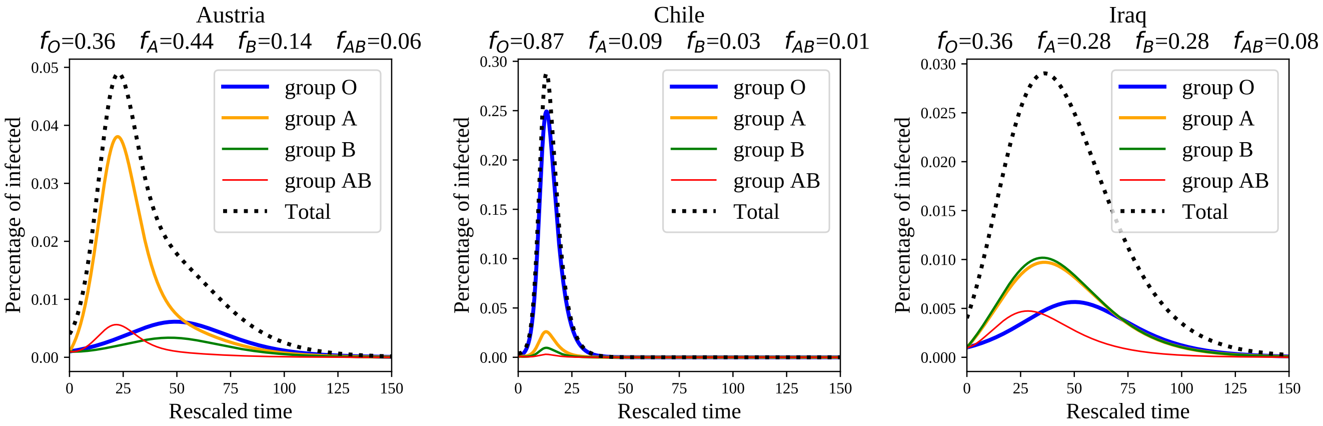

FIG. 2: **Example of numerical solution of the generalised SIR model for four sub-populations ($k = 4$) and for the infectivity matrix of Eq. (59).** The reported example is for $\rho = 0.3$, using real blood group frequencies registered in Austria, Chile and Iraq. Solid lines represent the fraction of infected individuals as a function of time (arbitrary units). The dashed lines represent the total fraction of infected people summing those of the four sub-populations.

and the sum rule

$$\sum_{i=1}^{k} f_i = 1 \tag{62}$$

allows one to work out the short time expansion for the infected person fraction on each of the k sub-populations:

$$y_i(\tau) = y_o f_i \, e^{\pi_i^{(k)}\tau} \qquad\qquad i = 1, ..., k \tag{63}$$

with

$$\pi_i^{(k)} = \sum_j W_{ij} f_j - \rho = (\mathbf{W} \cdot \bar{f})_i - \rho \doteq p_i^{(k)} - \rho, \tag{64}$$

as well as the total number of infected people:

$$y_T(\tau) = \sum_i y_i(\tau) = y_o \, e^{\pi_T^{(k)}\tau} \qquad\qquad i = 1, ..., k \tag{65}$$

with

$$\pi_T^{(k)} = \sum_i \sum_j f_i W_{ij} f_j - \rho = \bar{f} \cdot \mathbf{W} \cdot \bar{f} - \rho \doteq p_T^{(k)} - \rho \tag{66}$$

which implicitly define $p_i^{(k)}$ and $p_T^{(k)}$. This simple expression for the inverse of the characteristic time of the infection at its initial stage is the sum of two terms: , $\pi_T^{(k)} = p_T^{(k)} - \rho$.

The first one,

$$p_T^{(k)} \doteq \bar{f} \cdot \mathbf{W} \cdot \bar{f} \tag{67}$$

depends only on the abundance of the sub-population ($\bar{f}$) and on the infection rules ($\mathbf{W}$), the second ($\rho = \gamma/\beta$) on the overall recovery and infection rates of populations.

To study the global effect of the population composition on the progression of the infection, we concentrate on the term $p_T^{(k)}$ which acts as a "susceptibility".

It is worth to note that, for any k, the susceptibility is maximum when $\bar{f} = (0...0, 1, 0, ...0)$, $p_T^{(k)} = 1$, i.e. when one sub-population fraction dominates, while it minimum when the sub-populations are all of the same size: $\bar{f} =$

$(1/k, 1/k...1/k)$. In the latter case, for the infection rules reported before, $p_T^{(k)} = (3/4)^{(k/2)}$. Thus the susceptibility decreases on increasing the number of sub-populations and decreases on equalizing their abundances. Infection rules and compositions of the population are expected to shape the infection dynamics along with the usual infection and recovering rates.

Finally, in Figure 2, we show the solutions for three different sets of blood abundances. We took real data from three different countries. As one can see, cases in which most of the population has the same blood type (e.g. in Chile), the infection propagates very rapidly, infecting more individuals than in cases where blood groups are more homogeneously distributed, as for Iraq data. It is also interesting to note that as a consequence of the infection rules, the O group population get infected on much longer times with respect to the AB population, whose number of infected individuals rapidly increases. Overall, those effects produce different curves if we look at the total number of infected (black dotted lines). However, it is not possible to compare those predictions with data as each country implemented different infection-containment strategies, at different times, thus altering the course of the infection after the initial exponential regime.

II. FITTING TO INDIVIDUAL COUNTRY DATA

We collected data of the contagion by country from World Health Organization (WHO) Coronavirus Disease (COVID-19) Dashboard on date 12th of June 2020[3]. To ensure statistical reliability, we selected only countries that had registered at least 2000 positive cases from the start of the epidemic. Requiring also to know the frequencies of both ABO and $RhD\pm$, we ended up with 78 countries, whose information is reported in Table II.

In particular, the WHO data reports the number of new infections per day, $\dot{D}(t)$. From this quantity, we easily obtain the cumulative number of people that have been infected as a function of time, $D(t) = \int_o^t \dot{D}(t')dt'$. The cumulative, rather than directly with $\dot{D}(t)$ allows us to work on cleaner and more solid data because the day-by-day fluctuations are averaged out in the long run. Coming back to the model, at short times the cumulative $D_T(\tau)$ is directly related to $y_T(\tau)$, which short-time expansion is reported in Eq. (65). Overall, the COVID19 infection is characterized by low mortality but a high infectivity rate together with both long incubation and recovery periods. These conditions assure that limiting our analysis to the early stages of the infection, the cumulative can be regarded as a good proxy for the number of infected. For each country reported in Table 2 of the Main Text, we manually selected the time interval corresponding to the initial stage of the infection. In that range, we performed an unsupervised fit using the function:

$$Y(t) = \begin{cases} m_0 t & \text{if} \quad t < t_0 \\ m_0 t_0 + A\left(e^{m(t-t_0)} - 1\right) & \text{if} \quad t > t_0 \end{cases} \tag{68}$$

with t_o, m_o, m and A as free parameters. In this expression, A is a scaling factor and m represents the inverse of the characteristic time of the infection exponential growth, which is the quantity that we are looking at. The linear term, observed at the very beginning of the infection curve of different (not all) countries, can be rationalized by assuming that at the very early stage of the infection, people arrive from other countries, spreading the virus before the real exponential growth appears. The constant m_0 is the "arrival rate" of infected people (which is reasonable to assume constant in time), and we do not expect any correlation between m and m_0, because m is an intrinsic characteristic of the population, and m_0 only depend on the arrival from abroad. Besides the specific meaning of the different parameters, our aim is to derive the value of m, to compare this value with the prediction of the model $\pi_T^{(k)}$.

Figure 3 shows examples of cumulative growth curves (blue lines) of the number of infected people found in different countries of Europe, Asia and America. Red lines represent the best fit solution using Eq. 68 as fitting function.

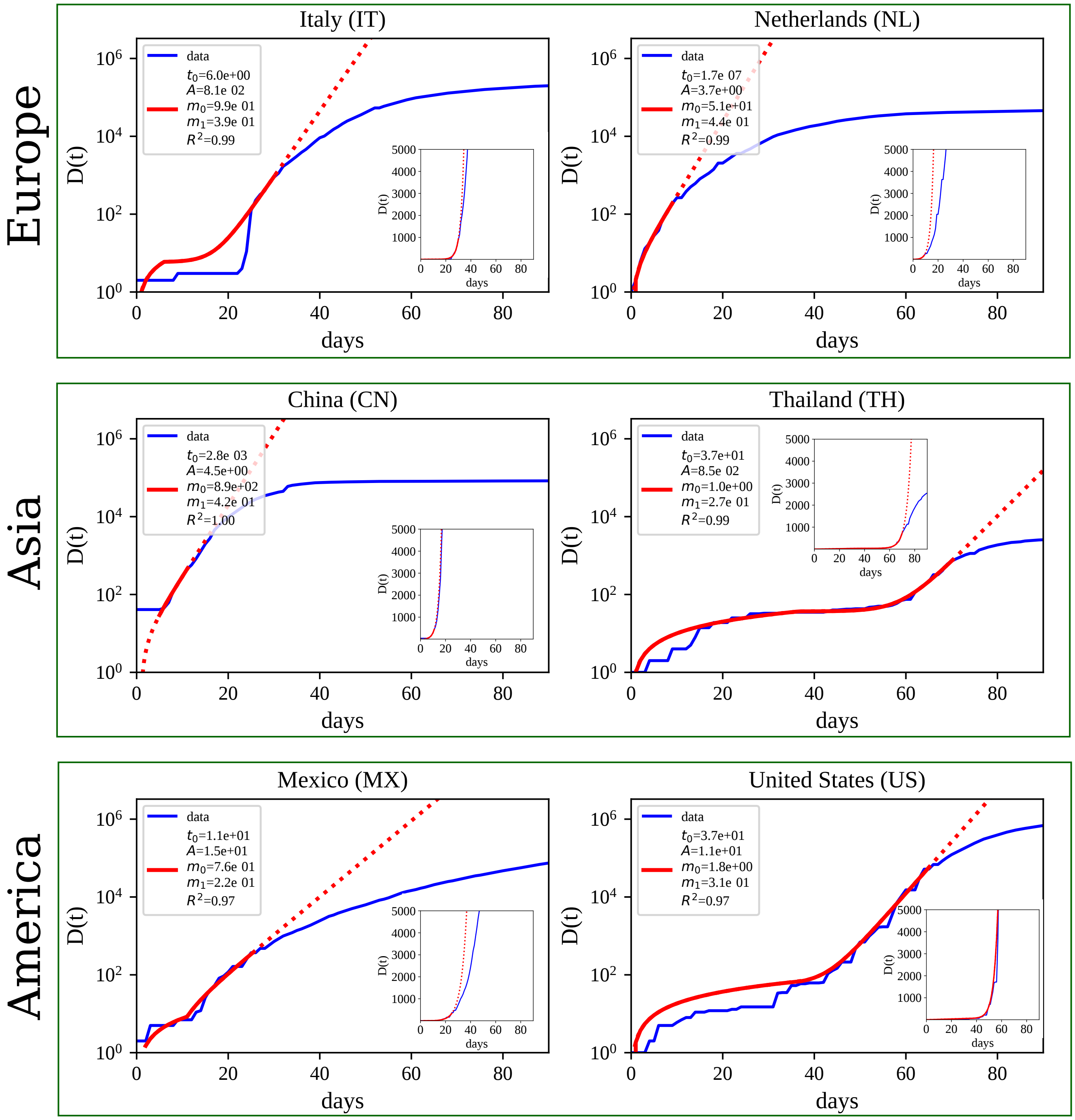

FIG. 3: Cumulative growth curves (blue lines) in logarithmic scale of the number of infected people found in different countries of Europe, Asia and America. Red lines represent the best fit solution using Eq. 68 as fitting function. The inset shows the same curves but in linear scale.

[1] R M Anderson, B Anderson, and R M May. *Infectious Diseases of Humans: Dynamics and Control*. Dynamics and Control. OUP Oxford, 1992.

[2] T Harko, F S N Lobo, and M K Mak. Exact analytical solutions of the susceptible-infected-recovered (SIR) epidemic model and of the SIR model with equal death and birth rates. *Applied Mathematics and Computation*, 236:184–194, June 2014.

[3] Geneva: World Health Organization. Who coronavirus disease (covid-19) dashboard, 2020. [Online; downloaded the 12th of June 2020].
