# Supplementary figures and images for: Does blood type affect the COVID-19 infection pattern?
Source: PLoS One. 2021 May 13;16(5):e0251535. doi: 10.1371/journal.pone.0251535 (PMC8118288; doi:10.1371/journal.pone.0251535)

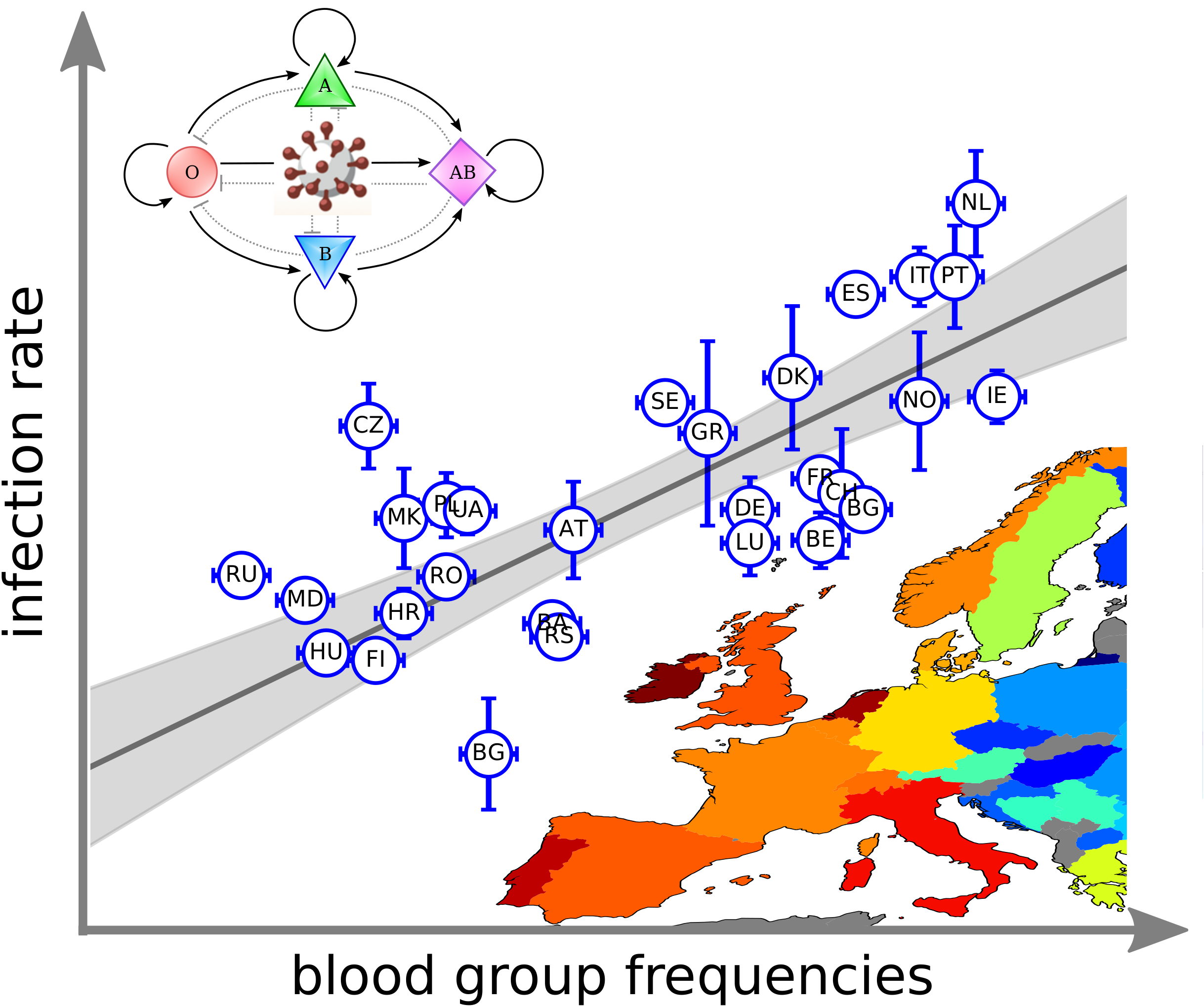

Supplement: S1 Graphical abstract — (TIF) [file pone.0251535.s002.tif]
